# A blood-free modeling approach for the quantification of the blood-to-brain tracer exchange in TSPO PET imaging

**DOI:** 10.3389/fnins.2024.1395769

**Published:** 2024-07-22

**Authors:** Lucia Maccioni, Carranza Mellana Michelle, Ludovica Brusaferri, Erica Silvestri, Alessandra Bertoldo, Julia J. Schubert, Maria A. Nettis, Valeria Mondelli, Oliver Howes, Federico E. Turkheimer, Michel Bottlaender, Benedetta Bodini, Bruno Stankoff, Marco L. Loggia, Mattia Veronese

**Affiliations:** ^1^Department of Information Engineering, University of Padova, Padova, Italy; ^2^Paris Brain Institute, ICM, CNRS, Inserm, Sorbonne Université, Paris, France; ^3^Athinoula A. Martinos Center for Biomedical Imaging, Department of Radiology, Massachusetts General Hospital, Harvard Medical School, Charlestown, MA, United States; ^4^Computer Science and Informatics, School of Engineering, London South Bank University, London, United Kingdom; ^5^Padova Neuroscience Center, University of Padova, Padova, Italy; ^6^Institute of Psychiatry, Psychology and Neuroscience (IoPPN), King’s College London, London, United Kingdom; ^7^BioMaps, Service Hospitalier Frédéric Joliot CEA, CNRS Inserm, Université Paris-Saclay, Orsay, France

**Keywords:** BBB, IDIF, kinetic modeling, PET, TSPO, neuroinflammation

## Abstract

**Introduction:**

Recent evidence suggests the blood-to-brain influx rate (*K_1_*) in *TSPO PET* imaging as a promising biomarker of blood–brain barrier (*BBB*) permeability alterations commonly associated with peripheral inflammation and heightened immune activity in the brain. However, standard compartmental modeling quantification is limited by the requirement of invasive and laborious procedures for extracting an arterial blood input function. In this study, we validate a simplified blood-free methodologic framework for *K_1_* estimation by fitting the early phase tracer dynamics using a single irreversible compartment model and an image-derived input function (*1T1K-IDIF*).

**Methods:**

The method is tested on a multi-site dataset containing 177 *PET* studies from two *TSPO* tracers ([^11^C]PBR28 and [^18^F]DPA714). Firstly, *1T1K-IDIF K_1_* estimates were compared in terms of both bias and correlation with standard kinetic methodology. Then, the method was tested on an independent sample of [^11^C]PBR28 scans before and after inflammatory interferon-*α* challenge, and on test–retest dataset of [^18^F]DPA714 scans.

**Results:**

Comparison with standard kinetic methodology showed good-to-excellent intra-subject correlation for regional *1T1K-IDIF-K_1_* (*ρ_intra_* = 0.93 ± 0.08), although the bias was variable depending on *IDIF* ability to approximate blood input functions (0.03–0.39 mL/cm^3^/min). *1T1K-IDIF-K_1_* unveiled a significant reduction of *BBB* permeability after inflammatory interferon-*α* challenge, replicating results from standard quantification. High intra-subject correlation (*ρ* = 0.97 ± 0.01) was reported between *K_1_* estimates of test and retest scans.

**Discussion:**

This evidence supports *1T1K-IDIF* as blood-free alternative to assess *TSPO* tracers’ unidirectional blood brain clearance. *K_1_* investigation could complement more traditional measures in *TSPO* studies, and even allow further mechanistic insight in the interpretation of *TSPO* signal.

## 1 Introduction

Inflammatory processes are involved in the pathophysiology of a wide spectrum of brain disorders (Albrecht et al., 2016; Guzman-Martinez et al., 2019) The prolonged activation of microglia and astrocytes, the resident immune cells in the brain, and the infiltration of peripheral immune cells in the brain parenchyma have been associated with the onset and/or progression of neurodegenerative disorders - such as Alzheimer’s disease, Parkinson’s disease and multiple sclerosis -, neuropsychiatric disorders - such as schizophrenia and depression -, but also stroke, and chronic pain (Loggia et al., 2015; Bauer and Teixeira, 2019; Goldsmith et al., 2019; Nutma et al., 2019; Dimitrova-Shumkovska et al., 2020; Han et al., 2021).

The growing interest in neuroinflammation has motivated the introduction in the past decade of a substantial number of imaging biomarkers designed to detect *in vivo* brain inflammation (Vicente-Rodríguez et al., 2021).

Positron Emission Tomography (*PET*) imaging represents a powerful tool for the *in vivo* characterization of neuroinflammatory processes (Turkheimer et al., 2015; Werry et al., 2019; Jain et al., 2020). The majority of *PET* imaging studies of neuroinflammation utilize radiotracers targeting the 18 kDa translocator protein (*TSPO*), which is expressed in activated microglia, and also in astrocytes and endothelial cells, and is upregulated in neuro-immune responses (Jain et al., 2020). Despite its limitations (Vivash and O’Brien, 2016; De Picker and Haarman, 2021; Zhang et al., 2021; Nutma et al., 2023), *TSPO PET* is currently the most specific method for imaging neuroinflammation in the living human brain.

**Figure 1:**
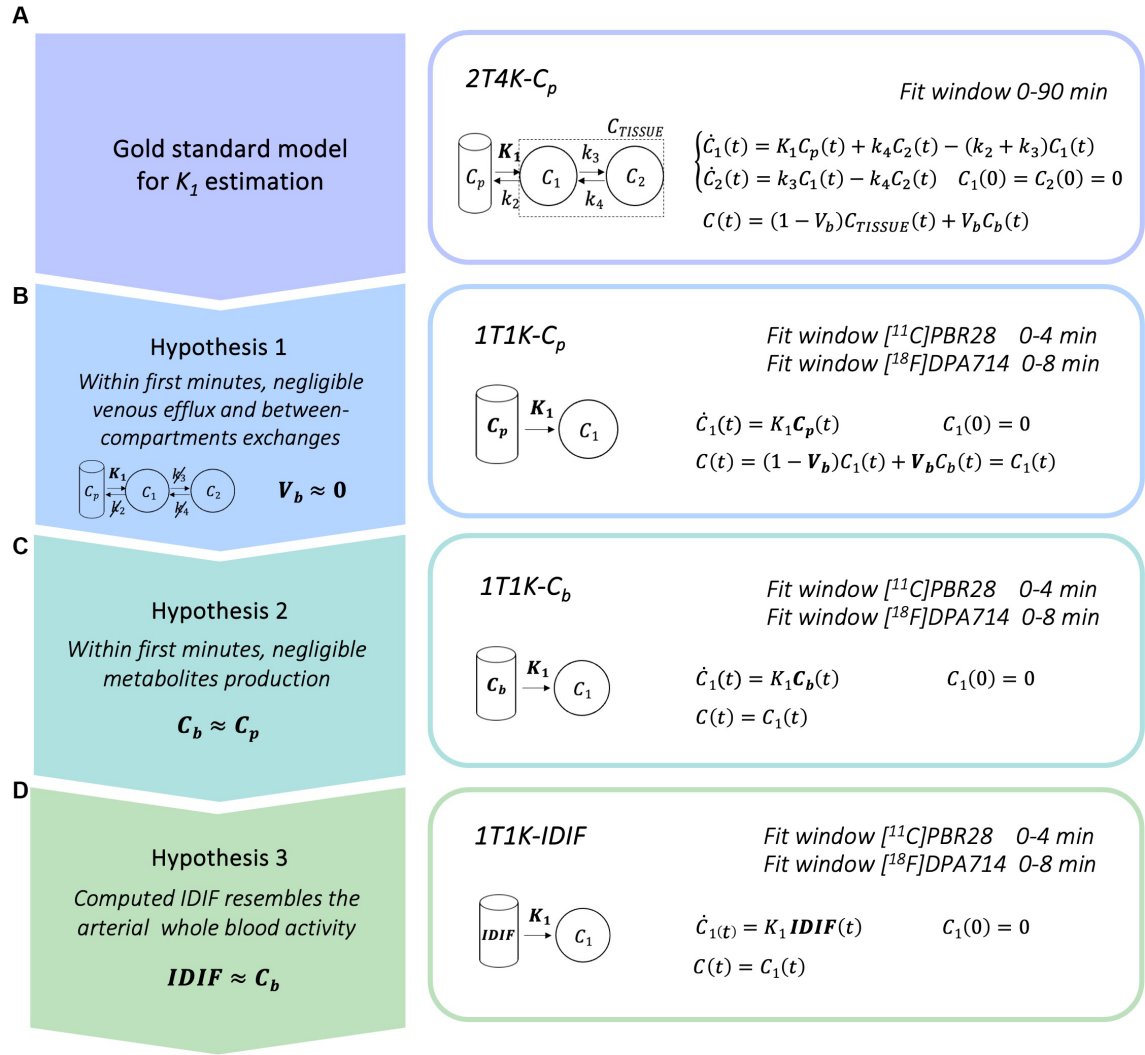
Simplified blood-free methodological framework for *K_1_* estimation. Panel **A** show the gold standard compartmental model for kinetic modeling of *TSPO* radiotracers; panels **B–D** show the simplified models for the *K_1_* estimation derived when considering a limited time window for model fitting [*C_p_* = arterial parent plasma input function, *C_b_* = arterial whole blood input function, *IDIF* = image derived input function].

Compartmental modeling is the standard methodology for the quantification of dynamic *PET* data: it provides a mathematical description of the kinetic of the radiotracer within the target tissues as a function of the tracer concentration in the plasma over time. Plasma tracer activity commonly defines the input function of the model, while the model parameters describe the tracer kinetics (Bertoldo et al., 2014). In the case of *TSPO PET* tracers, the most widely used kinetic model is composed of two reversible compartments and defined by 4 rate constants (i.e., *K_1_*, *k_2_*, *k_3_*, *k_4_*; Turkheimer et al., 2015; Wimberley et al., 2021; Figure 1A), which can be extended with the inclusion of a vascular component (Rizzo et al., 2014). If the input is known, the model parameters can be estimated by fitting the model to the measured time activity curves (*TACs*). Model parameters are then combined to quantify metrics of interest such as the volume of distribution (*V_T,_*; Innis et al., 2007), which is widely employed in *TSPO PET* studies as a proxy of the density of *TSPO* (Rizzo et al., 2014; Marques et al., 2021).

Among these parameters, is the influx rate constant *K_1_* (*mL/cm^3^/min*). This metric denotes the rate at which the tracer crosses the blood–brain barrier (*BBB*) from plasma and, according to Fick’s law, can be expressed as the product of cerebral blood flow and tracer *BBB* extraction fraction (Renkin, 1959; Crone, 1963).

The *BBB* represents the main regulatory interface between the central nervous system and the immune system (Erickson et al., 2012). A growing body of evidence indicates its modulation or disruption as a common hallmark of neurodegeneration in neuroinflammatory conditions (Takata et al., 2021): a likely protective reduction of *BBB* permeability has been suggested as a response to mild peripheral cytokine levels (Turkheimer et al., 2023), while *BBB* leakage has been demonstrated in several brain disorders, such as multiple sclerosis (Kirk et al., 2003), neurodegenerative disorders (Zlokovic, 2008), and chronic inflammatory pain (Brooks et al., 2005).

The reasons above have motivated a recent interest in the investigation of *K_1_* alterations. On the other hand, kinetic modeling in *TSPO PET* imaging is hampered by logistical considerations linked to the necessity to accurately quantify the metabolite-corrected blood input function. Blood input function requires invasive arterial sampling, which can cause discomfort to the participants, and laborious procedures for the counting of blood and plasma tracer concentrations, the extraction and quantification of radio-metabolites as well as the measurement of the tracer free plasma fraction (*fp*), i.e., the fraction of the tracer not bound to plasma proteins (Tonietto et al., 2016, 2019).

Many alternative blood-free approaches have been proposed in the past years to overcome the practical limitations of arterial blood-based methods. Among these is the use of an image-derived input function (*IDIF*). However, the actual use of the *IDIF* approach in research studies has been limited by several issues, primarily the challenge of deriving from the image the information on the tracer parent plasma fraction (*PPf*; Zanotti-Fregonara et al., 2011a, b; Volpi et al., 2023).

In this work, we propose a new non-invasive and simplified methodological framework for the computation of the rate *K_1_*, that consists of fitting a limited time window of the tracer kinetic after tracer injection (within 10 min), with a simplified model composed of a single irreversible compartment and a non-invasive *IDIF* (*1T1K-IDIF*). Validation was performed on two second-generation *TSPO PET* tracers, namely [^11^C]PBR28 and [^18^F]DPA714, on dynamic scans gathered from three different *PET* centers (the *Centre for Neuroimaging Sciences at King’s College London*, the *Athinoula A. Martinos Center of Biomedical Imaging*, *Massachusetts General Hospital*, and the *Paris Brain Institute, Sorbonne University*). Model evaluation encompassed three main steps: (1) comparison of *1T1K-IDIF* to standard-reference *2T4K-C_p_* kinetic modeling; (2) application of *1T1K-IDIF* approach to the study of *K_1_* alterations linked to peripheral inflammation on [^11^C]PBR28 *PET* scans for which a statistically significant reduction of the gray matter influx rate measured by the standard kinetic methodology was previously reported (Turkheimer et al., 2021); (3) evaluation of the reproducibility of *1T1K-IDIF* estimates on test–retest [^18^F]DPA714 *PET* data.

## 2 Materials and methods

### 2.1 Methodological framework

The current standard for the quantification of the influx rate constant for *TSPO PET* tracers is the use of the reversible two tissues compartmental model, with metabolite corrected plasma input function (*C_p_*), to fit the tissue whole-dynamic *TAC* (*2T4K-C_p_*; Figure 1A).

Here, we propose a simplified quantification approach consisting of the estimation of the *K_1_* kinetic parameter via fitting of the first minutes (within 10 min) of the tracer kinetic in the tissue. This methodological framework was readapted from a previous research project performed in the context of β*-*amyloid *PET* imaging (Silvestri, 2018). With the aim of deriving a completely non-invasive procedure, the method relies on 3 different assumptions:

1. Within the first minutes after the injection, it is reasonable to assume that exchanges between the first and the second compartment and the venous efflux have not yet occurred, or if it is so, that their effect on the dynamics is negligible. In this framework, the kinetic of the tracer is mostly reflecting the tracer influx from blood to the brain parenchyma and the two tissues reversible compartment model can be reduced to a model with only one irreversible compartment. The model equation can be further simplified through the deletion of the parametrization of the fraction of blood volume (*V_b_* = 0), which brings to the definition of the final linear model for the estimation of *K_1_* parameter (*1T1K-C_p_*, Figure 1B). Of note, the assumption of null *V_b_*, while commonly applied in the context of brain *PET* parametric imaging (voxel resolution), represents a relevant approximation in the case of region-of-interest (*ROI*) analysis.
2. In a limited time-window after tracer injection, the production of metabolites from the parent radiotracers is still limited (Tonietto et al., 2016; Peyronneau et al., 2023). In the case of many *PET* tracers, it is possible to identify a specific time window after tracer injection in which the impact of the metabolites on the *PPf* is still negligible (<10%) as compared to the total parent. If the concentration of metabolites is negligible, we can also assume an even tracer distribution between red blood cells and plasma that is generally, but not solely, driven by polar radioactive molecules. Hence, we can assume the whole blood tracer concentration (*C_b_*) as a reasonable approximation of the parent plasma tracer concentration and use it as the input function of the model (*1T1K-C_b_*; Figure 1C).
3. In this framework, by the adoption of a robust protocol for image-derived input function extraction and by assuming that the computed *IDIF* is a good approximation of the *C_b_*, it can be adopted as the input function of the compartmental model (*1T1K-IDIF*; Figure 1D), providing a completely blood sampling free method for the estimation of the *K_1_* parameter. *IDIF* from brain *PET* protocols is known to generally represent a poor alternative to *C_p_*, especially in the case of low spatial and temporal resolution of dynamic images of traditional brain *PET* scanners (Volpi et al., 2023). This third assumption is thus expected to be the most penalizing, with performances of the *K_1_* estimation highly dependent on the ability to retrieve a reliable estimate of the *IDIF.*

### 2.2 Study participants and data acquisition

Five datasets were adopted in this study. In total, 159 [^11^C]PBR28 PET imaging scans from two independent research centers (*King’s College London (KCL) and Athinoula A. Martinos Center for Biomedical Imaging, Massachusetts General Hospital (MGH)*) and 18 [^18^F]DPA714 scans from the *Paris Brain Institute of Sorbonne University (ICM)* were included.

All *PET* imaging sessions were acquired with a continuous dynamic acquisition, from 0 to 90 min after a bolus injection of the tracer. For all participants (but one subject from the *ICM* [^18^F]DPA714 dataset), arterial blood data were sampled via radial artery catheter at the time of the scan and corrected for metabolites. *PET* data reconstruction varied across imaging sites and scanner types, but all included correction for random and scattered coincidences and tissue attenuation.

Given the genetic *rs6971* polymorphism of the *TSPO* gene, which conveys different affinity profiles for *TSPO* radioligands [high affinity binder (*HAB*), mixed affinity binder (*MAB*), or low affinity binder (*LAB*; Wimberley et al., 2021)], all participants were genotyped before scanning, and only *HABs* and *MABs* were retained for further analysis.

With the purpose of tissue segmentation and *ROIs* parcellation, structural T1-weighted (*T1w*) Magnetic Resonance (*MR*) images were also acquired for each participant (simultaneously to *PET* acquisition at *MGH*, in separate visits at *KCL* and *ICM*). Studies were approved by local ethics committees and institutional review boards prior to start, and all participants provided informed consent after reading a full description of the study.

#### 2.2.1 *KCL* [^11^C]PBR28 data

A total of 108 dynamic [^11^C]PBR28 *PET* and *MR* images were shared by *KCL*. Available data included a dataset of 94 scans (*Dataset 1*) collected in healthy volunteers and psychiatric patients gathered from *KCL* historical database (Age: 34 ± 14 years; Sex: 62 male and 32 female; Genotype: 63 HABs and 31 MABs; Clinical population: 65 healthy controls, 14 subjects at ultra-high risk of psychosis, 15 patients with schizophrenia). A second dataset (*Dataset 2*) was composed of [^11^C]PBR28 dynamic *PET* and *MR* scans on 7 healthy controls (Age: 30 ± 6 years; Sex: 7 male; Genotype: 7 HABs), who each received scans before and 24 h after an immune challenge performed via subcutaneous injection of *IFN-α* 2a (Roferon-A 3 million IU/0.5 mL solution for injection).

Details on participants and data acquisition for the two datasets can be found in Bloomfield et al. (2016); Dahoun et al. (2019); and Nettis et al. (2020). In brief, all acquisition protocols included an initial low-dose computer tomography (*CT*) scan, acquired for attenuation and scatter correction, using a *Siemens Biograph^™^ TruePoint^™^ PET·CT scanner* (*Siemens Medical Systems, Germany*; transaxial field of view (*FOV*): 60.5 cm, axial *FOV*: 16.2 cm), followed by a 90 min dynamic *PET* scan after a bolus injection of [^11^C]PBR28 (Injected Dose: 329.30 ± 27.43 MBq). Dynamic *PET* data were binned into 26 frames (durations: 8 × 15 s, 3 × 1 min, 5 × 2 min, 5 × 5 min, 5 × 10 min), reconstructed using filtered back projection, with a 5 mm isotropic Gaussian smoothing, and corrected for random noise, attenuation, and scatter effects. Radio-pharmaceutical preparation acquisition protocol was consistent for all the studies. *T1w MR* brain scan data were collected using a *Siemens 3-T MR* scanner on either a *Siemens Tim Trio* or *Siemens MAGNETOM Verio*.

Arterial blood data were sampled using the combination of an automatic continuous sampling system of the whole blood activity for the first 15 min of each scan, and a series of discrete blood samples, manually taken at 5, 10, 15, 20, 25, 30, 40, 50, 60, 70, 80, and 90 min. Manual samples were centrifuged and used to determine the plasma over blood activity ratio (*POB*). Samples taken at 5, 10, 20, 30, 50, 70, and 90 min were also analyzed using radio-high performance liquid chromatography (*HPLC*) to calculate the *PPf*. The MIAKAT software (*MIAKAT™*)^1^ was adopted for blood data processing: subjects’ *C_b_* were defined by combining and calibrating continuous and discrete whole blood data; a linear and a sigmoid model were, respectively, fitted over *POB* and *PPf* discrete samples and applied for the definition of uncorrected plasma activity (*C_p_uncor_*) and *C_p_* for each subject.

#### 2.2.2 MGH [^11^C]PBR28 data

*MGH* dataset (*Dataset 3*) included dynamic [^11^C]PBR28 *PET/MR* images from 51 individuals [Age: 55 ± 16 years; Sex: 27 male and 24 female; Genotype: 31 HABs and 20 MABs; Clinical population: 10 Healthy Controls, 41 subjects with chronic musculoskeletal pain (low back pain, *N* = 26; knee osteoarthritis, *N* = 15)] simultaneously collected on a *Siemens Biograph mMR* whole-body *PET/MR* scanner (transaxial *FOV*: 59.4 cm; axial *FOV*: 25.8 cm). Full details on participant inclusion criteria and data acquisition can be found in original references (Weerasekera et al., 2021; Morrissey et al., 2023).

For all these studies dynamic PET data were acquired for a time period of 0–90 min after a bolus injection of [^11^C]PBR28 (Injected Dose: 523.60 ± 48.66 MBq), binned into 27 frames (duration: 8x10s, 3x20s, 2x30s, 1x1min, 1x2min, 1x3min, 8x5min, 3x10min) and reconstructed using the Ordered Subset Expectation Maximization (OSEM) with four iterations, 21 subsets, and a 3 mm Full Width Half Maximum (FWHM) Gaussian smoothing. Structural *T1w* images were used for the generation of attenuation correction maps using an in-house developed magnetic resonance-based approach (Izquierdo-Garcia et al., 2014).

Blood samples were collected at 5, 10, 20, 30, 50, 70, and 90 min post radiotracer injection. In 16 subjects, arterial blood processing was performed using a *HyperSep C18* solid extraction cartridge for separation of radio-metabolites from parent radiotracer; in 36 subjects, *HPLC* was used instead. For each scan, *C_p_uncor_* was obtained from collected blood samples and corrected for metabolites to compute the *C_p_*. *C_b_* was not available for these participants.

#### 2.2.3 ICM [^18^F]DPA714 data

[^18^F]DPA714 dynamic *PET* and *MR* scans acquired on 10 healthy volunteers at *SFHJ, CEA, Orsay,* were shared by the *ICM* center. Details on acquisition protocol are available in García-Lorenzo et al. (2018) and Lavisse et al. (2015).

For all participants, after a transmission scan using a 137Cs point source, a [^18^F]DPA714 slow bolus was intravenously injected over 1 min (Injected Dose: 203.05 ± 25.41 MBq) and subjects underwent dynamic *PET* scans in a high-resolution research tomograph (*HRRT, Siemens, Knoxville, TN, United States*; transaxial *FOV:* 31.2 cm, axial *FOV:* 25.5 cm) *at SHFJ-CEA, Orsay*. PET images were corrected for attenuation, random and scattered coincidences, reconstructed with the iterative ordered-subset expectation maximization (Ordinary Poisson [OP]-OSEM) 3D method, and binned into 27 time frames (durations: 6 × 1 min, 7 × 2 min, 14 × 5 min). A 3D Gaussian kernel with 2 mm FWHM was used as a point-spread function correction. Each participant underwent *T1w MR* imaging, performed with either a *Philips* (*Best, The Netherlands*) *Achieva 1.5 T MR* scanner or a *Siemens Trio 3 T* (*Erlangen, Germany*) scanner.

Eight of the participants underwent a second [^18^F]DPA714 dynamic *PET* to generate test–retest data (*Dataset 4*; Age: 44 ± 13 years; Sex: 2 male and 6 female; Genotype: 6 HABs and 2 MABs). For all participants, the second acquisition was performed between 5 and 67 days after the first visit, with a dose difference between the two acquisitions of 21.29 ± 18.95 MBq.

Metabolite-corrected and uncorrected plasma curves were generated only for 9 of the 10 subjects (*Dataset 5*; Age: 39 ± 13 years; Sex: 4 male and 5 female; Genotype: 5 HABs and 4 MABs). During *PET* scan acquisition, 21 sequential arterial blood samples were manually sampled and 7 of them (at 5, 10, 20, 40, 60, 70 and 90 min) analyzed as described in Peyronneau et al. (2023) to determine the *PPf*, *C_p_uncor_*, and *C_p_* activity for each subject. As for *MGH* dataset, *C_b_* was not available for these participants.

### 2.3 Image pre-processing

Data were analyzed by each individual site using different combinations of in-house codes and neuroimaging analysis software including *Statistical Parametric Mapping 8*,^2^ *FSL*^3^ and *MIAKAT*.

Despite these differences among the three centers, all pipelines included a step of motion correction of the dynamic *PET* data, the computation of integral *PET* images, the derivation of brain and gray matter masks from structural *MR* images—which was performed in all three cases using *FreeSurfer* package—and the registration of brain and tissues masks to the subject’s native *PET* space.

A neuroanatomical atlas was implemented and co-registered on the subject’s PET native space for the definition of *ROIs*. In detail, 46 cortical and 2 cerebellum *ROIs* (left and right hemisphere) defined by *CIC* neuroanatomical atlas version 2.0 (Tziortzi et al., 2011) were considered in the case of [^11^C]PBR28 data; 68 cortical and 1 cerebellum *ROI* defined by the *Desikan-Killany* atlas included with *FreeSurfer* (Desikan et al., 2006) were adopted for the [^18^F]DPA714 data. Mean regional *TACs* were computed for each subject.

### 2.4 Image-derived input function and blood data analysis

An image-derived input function was also calculated from each of the processed dynamic *PET* scans in the *PET* naïve space. The first step in the *IDIF* computation consisted of the segmentation of the arterial carotid siphons. This step was performed via intensity thresholding of early dynamic *PET* frames. In the case of *KCL* and *MGH* data, a preliminary crop mask was defined to delineate an anatomical area including the siphons, and then the siphons mask was defined by selecting the voxels belonging to the crop mask with the highest intensity in the early *PET* frames. In the case of *ICM* scans a manual segmentation of the internal carotids was already available. However, for consistency with the previous method, we decided to still perform a selection of the voxels belonging to the manual carotid mask with an intensity equal to or higher than 20% of the maximum value shown by the voxels in the so-defined *ROI*.

For each voxel, we computed the one-versus-all correlation of the voxels *TACs* and we heuristically selected 70% of voxels with the more correlated dynamics: this step was aimed at excluding the voxels with highly noisy dynamics. In the case of *MGH* data, where the number of selected voxels was highly variable among the subjects, a further step consisting of the selection of 30 voxels with the highest peak amplitude was performed; this step showed to guarantee a better description of the peak of the input function curve. *IDIF* was finally derived by averaging the *TACs* of all voxels resulting from the selection.

Since compartmental modeling assumes a noise-free input function, *IDIF* for each subject was finally fitted using the tri-exponential model, employing a linear regression for the rising part of the curve and a sum of three exponentials for the descending part of the curve (Parsey et al., 2000). Representative *IDIFs* are reported in Supplementary Figure 1.

Population *PPf* curves were computed for both [^11^C]PBR28 and [^18^F]DPA714. This step was aimed at investigating the presence of a time window after tracer injection in which the production of metabolites is negligible (i.e., *Hypothesis #2* of our methodological framework). Population curves were derived by averaging *PPf* data from [^11^C]PBR28 dynamic *PET* scans of 72 healthy subjects (*KCL Dataset 1* and *Dataset 2, pre INF-α*) and [^18^F]DPA714 *PET* scans of 9 healthy volunteers (*ICM Dataset 5*). Only in the case of *KCL* data, for which individual *POB* curves were available, population *POB* curves were computed from data of the 72 healthy subjects.

### 2.5 Model validation

All model fitting and statistical analyses were performed using *MATLAB* (*MathWorks*), although slightly different versions were adopted for the three *PET* centers: *Matlab2017b* for *KCL*, *Matlab2023a* for MGH and *Matlab2022b* for *ICM*. This discrepancy was the consequence of software and license availability on each site.

#### 2.5.1 Comparison with standard blood-based K_1_ estimates

As a first step in the validation, the performance of the proposed methodology in the estimation of *K_1_* parameter was evaluated by comparison to the standard reference *2T4K-C_p_* estimates. This step was performed on *Dataset 1,3,5*. *K_1_* regional estimates were computed for each subject’s *ROIs*, and bias introduced for each hypothesis of the proposed simplified method (Figure 1) was sequentially tested. Firstly, we tested whether the one irreversible compartment applied to early frames of the *PET* dynamics gave comparable results to those obtained with the full compartmental model and the true parent plasma adopted as input function (*1T1K-C_p_ K_1_*). Then, the effect of the assumptions of metabolites production and red blood cells uptake negligibility was tested by computation of the *K_1_* using the whole blood input function (*1T1K-C_b_ K_1_*). To note, information on the whole blood *TACs was* available only in the case of *KCL* scans. For *MGH* and *ICM* datasets the uncorrected plasma tracer concentration was adopted instead. Finally, regional *K_1_* parameters were computed with *IDIF* as input to the reduced model (*1T1K-IDIF K_1_*).

In all the cases, the *a priori* definition of the time window to use for model fitting was necessary. This choice was based on a trade-off between the opposite necessities to limit the model fit to a short time window, such that the model hypotheses were respected, and to include enough data points for model fitting. Ultimately the time window selection had to account for differences in the speed of the kinetics for the two employed tracers and in the frame binning of dynamic *PET* scans. The time window was selected for each tracer as the interval following tracer administration in which the parent plasma fraction was >90%. *ROIs TACs* were also visually inspected with the purpose of ensuring the applicability of *Hypothesis #1* of our methodological framework. This resulted in a time window of around 4 min for [^11^C]PBR28 and 8 min for [^18^F]DPA714.

Standard *2T4K-Cp K_1_* estimates were finally computed by fitting the whole 0-90 min tracer kinetic using a weighted non-linear least-square estimator with weights chosen optimally as


$$w_{ROI}\;\left(t_{i}\right)=\Delta t_{i}/C_{ROI}\left(t_{i}\;\right)$$

as suggested by Carson et al. (1983), where *t_i_* is the time instant, *Δt_i_* is the length of the scanning interval and *C_ROI_ (t_i_)* is the *ROI* average time activity at time *t_i_*. Both *C_p_* or *C_b_* input functions were corrected for delay (i.e., the variable time of appearance of tracer radioactivity in blood depending on the time required for the tracer to travel from the arterial sampling site to the region of interest; Iida et al., 1988).

To complete the corollary on *K_1_* analysis, assuming the cerebellum gray matter (*CER*) as a representative pseudo-reference region (Lyoo et al., 2015), the *K_1_* ratio


$$R_{1}=K_{1}/K_{1,CER}$$

was also computed for each method and each cortex *ROI*. The relative measure *R_1_* was introduced for completeness and consistency with previous *PET* studies where the *R_1_* measure can be used as a proxy of relative perfusion (Chen et al., 2015). However, it is important to highlight that the identification of a proper reference region remains not trivial, especially for TSPO studies, and disease specific (Albrecht et al., 2018).

Results obtained with each of the three simplified approaches (*1T1K-C_p_, 1T1K-C_b_, 1T1K-IDIF*), in terms of both *ROIs K_1_* and *R_1_* estimates, were compared to the respective standard *2T4K-C_p_* estimates by computation of bias and relative bias in absolute value (*bias* and *relBias*) and both intra- and between-subjects Pearson’s coefficients of correlation (*ρIntra, ρBetween*).

A multilinear regression model was finally estimated, describing the subjects’ mean *relBias* (averaged across *ROIs*) between the *1T1K-IDIF* and the *2T4K-C_p_ K_1_* estimates, in terms of subjects’ age, genotype classification, sex, *PET* center where the acquisition was performed, and the ratio between the area under the curve of *IDIF* and *C_p_* over the specific time window selected for fitting (*AUCratio*). Regression analysis was performed using the *fitglm* function (*Matlab2023a, Mathworks*). Predictors and response variables were rescaled to the same range [0 1]. In the case of categorical variables (genotype classification, gender, *PET* center), with *L* number of categories assumed by the categorical variable, the first category is assumed as the reference level, and the remaining *L – 1* are included as indicator variables in the model and treated as a single variable.

*1T1K-C_p_, 1T1K-C_b_, and 1T1K-IDIF* model fitting were repeated using different time intervals, ranging from 2 to 5 min in the case of [^11^C]PBR28 (*Dataset 1*) and from 3 to 9 min for [^18^F]DPA714 (*Dataset 5*). The sensitivity of model performance to the choice of the fitting window was investigated for each of the reduced models in terms of between-subjects and intra-subject correlation of *K_1_* and *R_1_* estimates and bias of *K_1_* estimates with respect to reference full model (*2T4K-C_p_*), and precision of *K_1_* estimates.

Additionally, *1T1K K_1_* estimation, with each of the three input functions, was repeated for [^11^C]PBR28 scans (*Dataset 1*) with the inclusion in the model of the parametrization of the fraction of blood volume (*1T1KV_b_*) and compared with the previous *1T1K-C_p_, 1T1K-C_b_* and *1T1K-IDIF K_1_* estimates obtained under the hypothesis of null *V_b_*.

Finally, the reversible one tissue compartment model (*1T2K*), with and without the *V_b_* parametrization, was tested and results of *1T1K* and *1T2K* fitting were compared both in terms of goodness of fit and consistency of *K_1_* estimates to standard *2T4K-C_p_ K_1_* estimates.

#### 2.5.2 IFN-α challenge-[^11^C]PBR28 scans

For each subject, regional cortex *2T4K-C_p_ K_1_* and *1T1K-IDIF K_1_* estimates were computed from both pre and post *IFN-α* challenge dynamic *PET* scans (*Dataset 2*) and averaged across the cortical *ROIs*. Distribution across subjects of pre and post mean cortex *K_1_* estimates were compared via a paired t-test following a test for normality (*Lilliefors test*).

#### 2.5.3 Test–retest reproducibility of K_1_ estimates-[^18^F]DPA714 scans

To test the reproducibility of the results, regional *1T1K-IDIF K_1_* estimates were computed for both test and retest scans on a pool of 8 healthy subjects (*Dataset 4*). *K_1_* estimates were computed for each subject (both test and retest scan) from mean *TACs* of 4 cortical brain lobes - frontal, temporal, occipital, and parietal -, insula, cerebellum, thalami, and brainstem. Pearson correlation coefficient (*ρ*) and mean root distance (*MRD*) were computed to compare *K_1_* obtained for test and retest scans.

## 3 Results

### 3.1 Population *PPf* and *POB* curves

**Figure 2:**
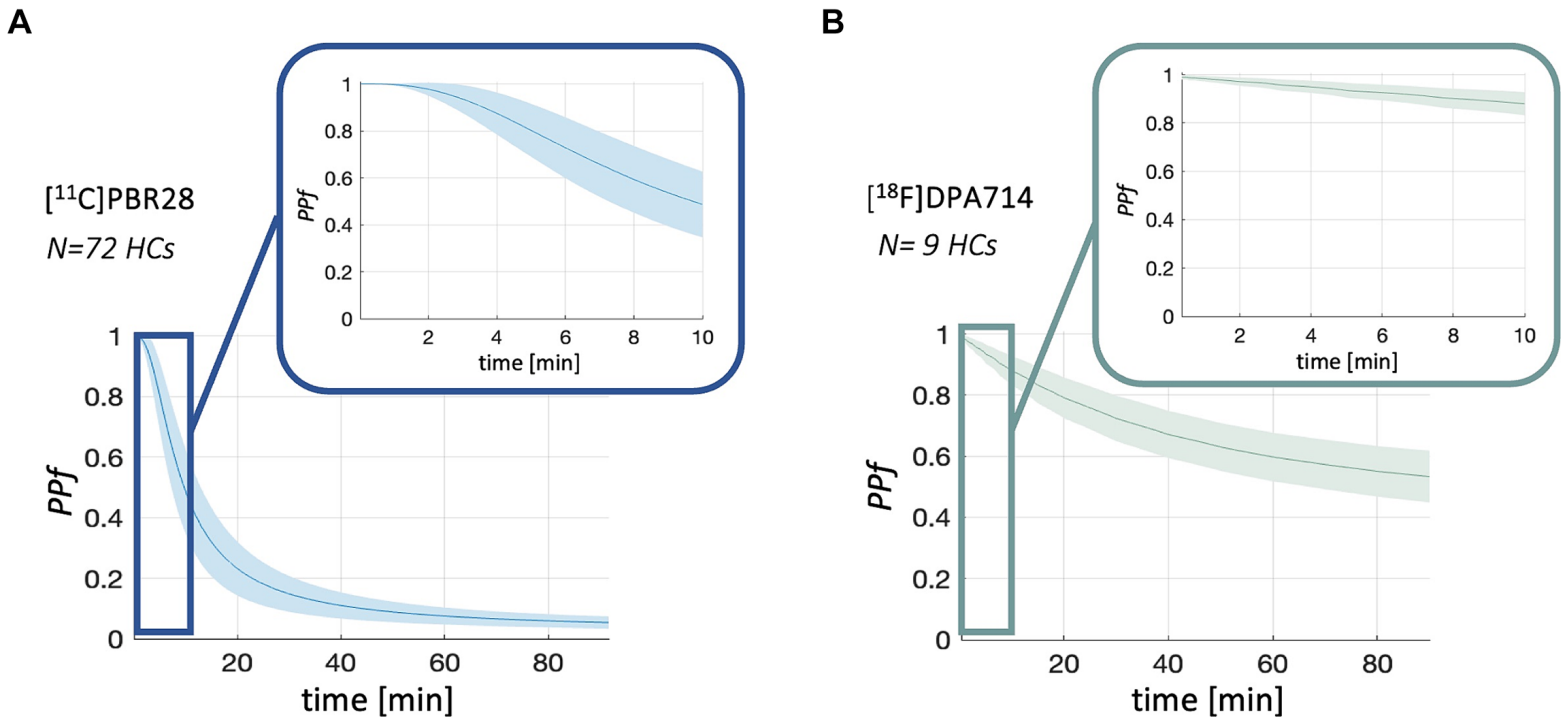
*PPf* population curves. Figure shows the mean (solid line) and standard deviation (shadowed band) of *PPf* curves for [^11^C]PBR28 (panel **A**) and [^18^F]DPA714 (panel **B**) in healthy controls (*HCs*) population.

**Figure 3:**
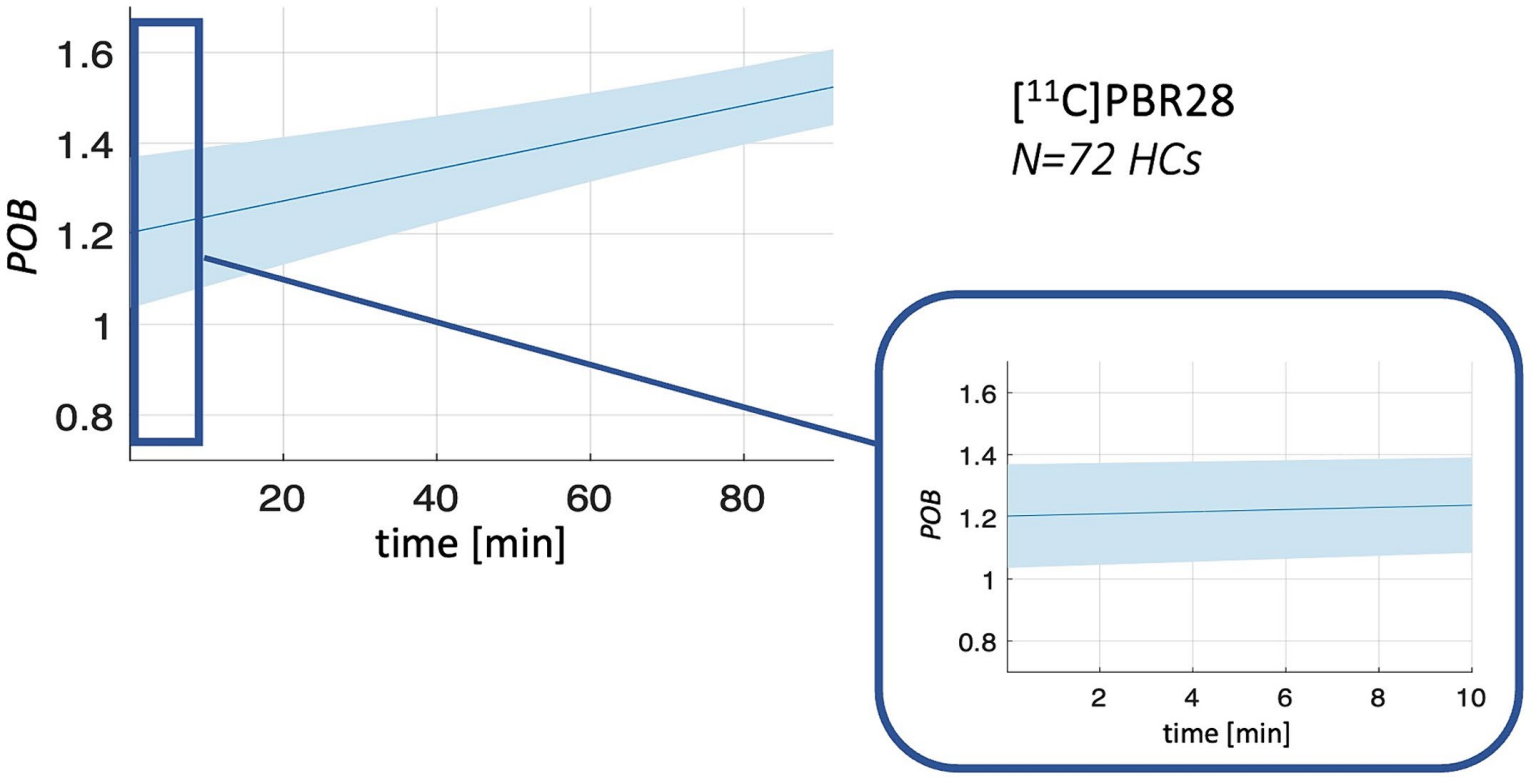
*POB* population curves. Figure shows the mean (solid line) and standard deviation (shadowed band) of *POB* curves for [^11^C]PBR28 in healthy controls (*HCs*) population.

Population *PPf* and *POB* curves are reported in Figures 2, 3. Average *PPf* curve for healthy populations reaches a value equal to 0.90 between 3 and 4 min for [^11^C]PBR28, while the 0.90 threshold is crossed at about 8 min in the case of [^18^F]DPA714.

### 3.2 Comparison with standard blood-based *K_1_* estimates

**Figure 4:**
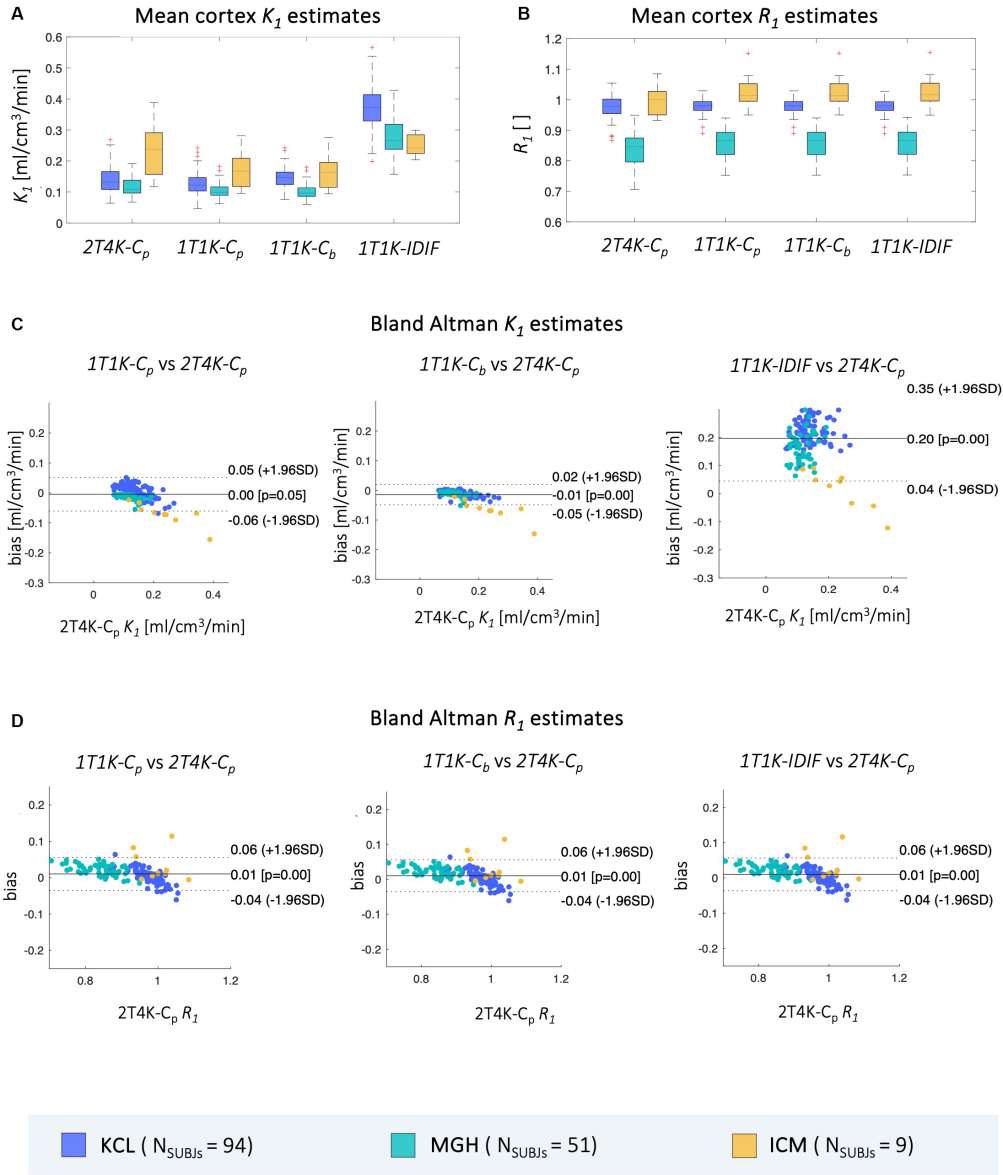
Comparison of mean cortex *K_1_* and *R_1_* estimates. Panel **A,B** show, respectively, the distribution across subjects of mean cortex *K_1_* and *R_1_* estimates computed via each of the aforementioned methods (*2T4K-C_p_*, *1T1K-C_p_*, *1T1K-C_b_*, *1T1K-IDIF*); panel **C,D** show the Bland Altman plots for the comparison between the results of each of the reduced methodologies (*1T1K-C_p_*, *1T1K-C_b_*, *1T1K-IDIF*) and the standard-reference *2T4K-C_p_* estimates for *K_1_* and *R_1_* respectively; results are shown in different colors for the three *PET* centers (*KCL*, *MGH*, *ICM*).

Results of mean cortex *K_1_* and *R_1_* estimates obtained from the datasets of the 3 *PET* centers are reported in Figure 4, together with Bland Altman plots comparing the mean cortex *1T1K-C_p,_ 1T1K-C_b,_* and *1T1K-IDIF* to the reference *2T4K-C_p_ K_1_* and *R_1_* estimates.

As for *K_1_* estimates, the highest bias is observed for the *1T1K-IDIF K_1_* when compared with the ground truth *2T4K-C_p_ K_1_*, with the biggest bias shown in the case of the *KCL* dataset and the lowest bias in the case of *ICM* data (*relBias [min max] %: relBias_KCL_ = [64.38324.57], relBias_MGH_ = [49.18310.50], relBias_ICM_ = [12.39 75.00]*). *relBias* from *2T4K-C_p_ K_1_* estimates results significantly reduced when *C_p_* (*relBias_KCL_ = [1.86 36.14], relBias_MGH_ = [3.15 35.07], relBias_ICM_ = [17.79 36.95]*) and *C_b_* (*relBias_KCL_ = [3.23 47.64], relBias_MGH_ = [4.00 37.60], relBias_ICM_ = [19.55 39.34]*) are adopted as input to the *1T1K* model, with an average *relBias* across subjects, respectively, of 10 and 16%. The relative bias for *R_1_* estimates is under 16% for all comparisons and centers.

**Figure 5:**
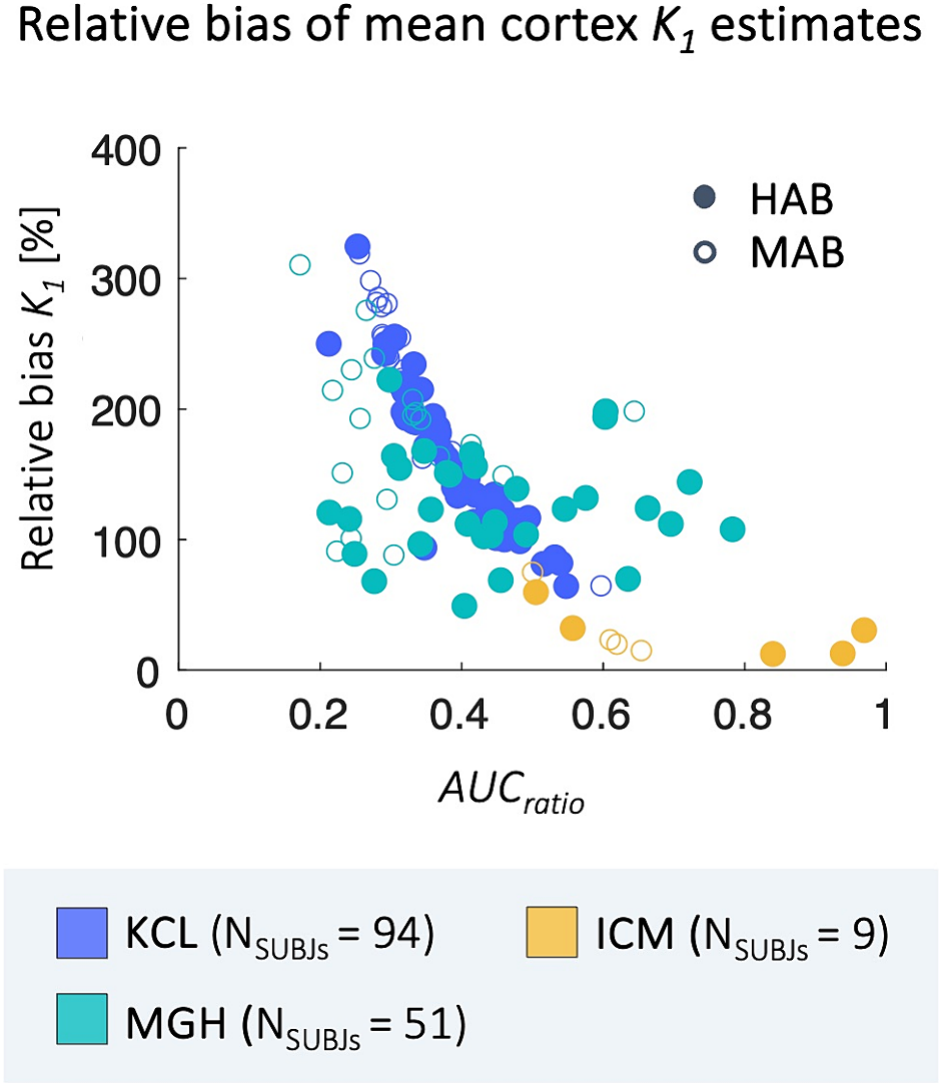
Correlation between *relBias* and *AUCratio.* Figure shows the correlation between subjects’ average *relBias* between the *1T1K-IDIF* and the *2T4K-C_p_ K_1_* and respective values of *AUCratio*; results are shown in different colors for the three *PET* centers (*KCL*, *MGH*, *ICM*) and with different shapes for *HAB* and *MAB* subjects.

Relative bias between *2T4K-C_p_* and *1T1K-IDIF K_1_* estimates shows a statistically significant correlation with the *AUCratio* (Figure 5); genotype classification and scan site also result as significant predictors in the regression model (*AUCratio*: *p* = 1.29*10^−8^, tstat = −6.03; genotype (*HAB* w.r.t. *MAB*): *p* = 0.0004, tstat = −3.66; *PET* center (*MGH* w.r.t. *KCL*): *p* = 0.04, tstat = −2.06; *PET* center (*ICM* w.r.t. *KCL*): *p* = 0.0003, tstat = −3.69; adjusted R^2^ = 0.51).

**Figure 6:**
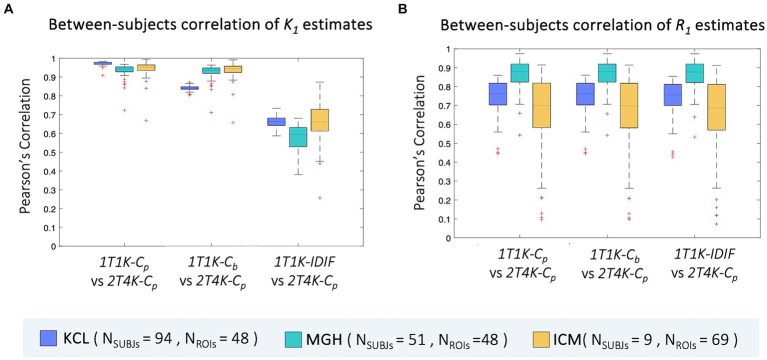
Between-subjects correlation of *ROIs K_1_* and *R_1_* estimates. Panel **A,B** show, respectively for the *ROIs K_1_* and *R_1_* estimates, the distribution of the between-subjects Pearson’s correlation coefficients between each of the reduced models (*1T1K-C_p_*, *1T1K-C_b_*, *1T1K-IDIF*) and the full model (*2T4K-C_p_*); results are shown in different colors for the three *PET* centers (*KCL*, *MGH*, *ICM*).

Between-subjects correlation analysis shows a similar pattern to bias, with the lowest Pearson’s coefficients reported when *IDIF* is adopted as the input function of the model. Comparable results are reported for the three sites (*ρ = mean ± standard deviation; ρBetween_KCL_ = 0.66 ± 0.03, ρBetween_MGH_ = 0.63 ± 0.06, ρBetween_ICM_ = 0.67 ± 0.11*). When comparing *1T1K-C_b_* with *2T4K-C_p_ K_1_* estimates, *KCL* dataset shows the lowest correlation (*ρBetween_KCL_ = 0.84 ± 0.02, ρBetween_MGH_ = 0.93 ± 0.04, ρBetween_ICM_ = 0.94 ± 0.05*) while comparable results among the three sites are shown for the between-subject correlation between *1T1K-C_p_* and *2T4K-C_p_* (*ρBetween_KCL_ = 0.97 ± 0.01, ρBetween_MGH_ = 0.93 ± 0.04, ρBetween_ICM_ = 0.94 ± 0.04*; Figure 6A). As for the *R_1_* estimates, model performance in terms of between-subject correlation resulted almost independent from the input function adopted (*ρBetween_KCL_ = 0.7 ± 0.1, ρBetween_MGH_ = 0.9 ± 0.1, ρBetween_ICM_ = 0.6 ± 0.3*; Figure 6B).

**Figure 7:**
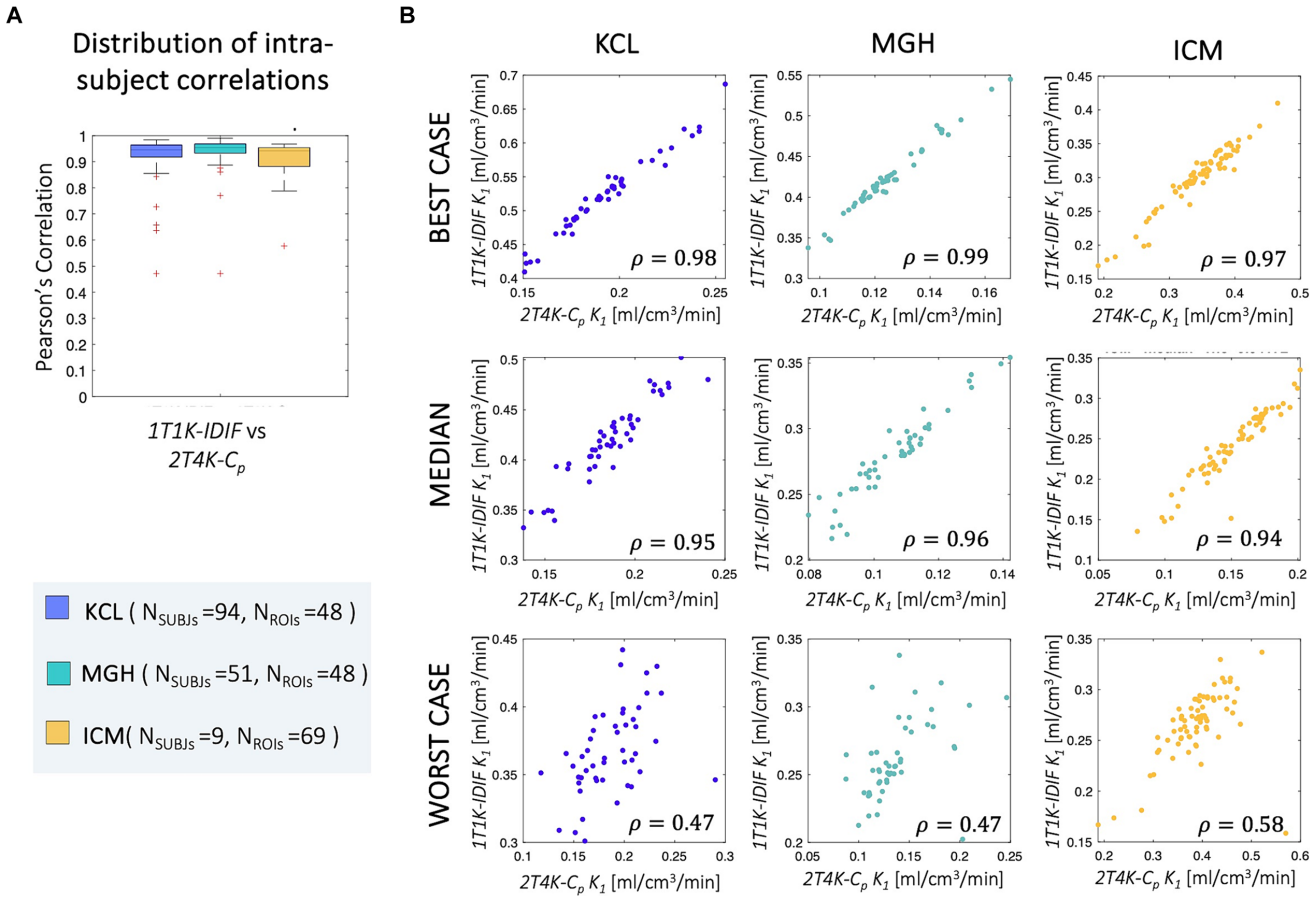
Intra-subject correlation of *ROIs K_1_* estimates. Panel **A** shows the distribution of the intra-subjects Pearson’s correlation coefficients between *1T1K-IDIF* and *2T4K-C_p_* regional *K_1_* estimates; panel **B** shows representative examples of the intra-subject correlation between *1T1K-IDIF* and *2T4K-C_p_* regional *K_1_* estimates; results are shown in different colors for the three *PET* centers (*KCL*, *MGH*, *ICM*).

Good-to-high intra-subject correlations were reported between the regional *K_1_* estimates obtained with the simplified and full model, independently from the input function adopted (*ρIntra_KCL_ = 0.93 ± 0.07, ρIntra_MGH_ = 0.94 ± 0.08, ρIntra_ICM_ = 0.89 ± 0.13*; Figure 7).

### 3.3 *IFN-α* challenge-[^11^C]PBR28 scans

**Figure 8:**
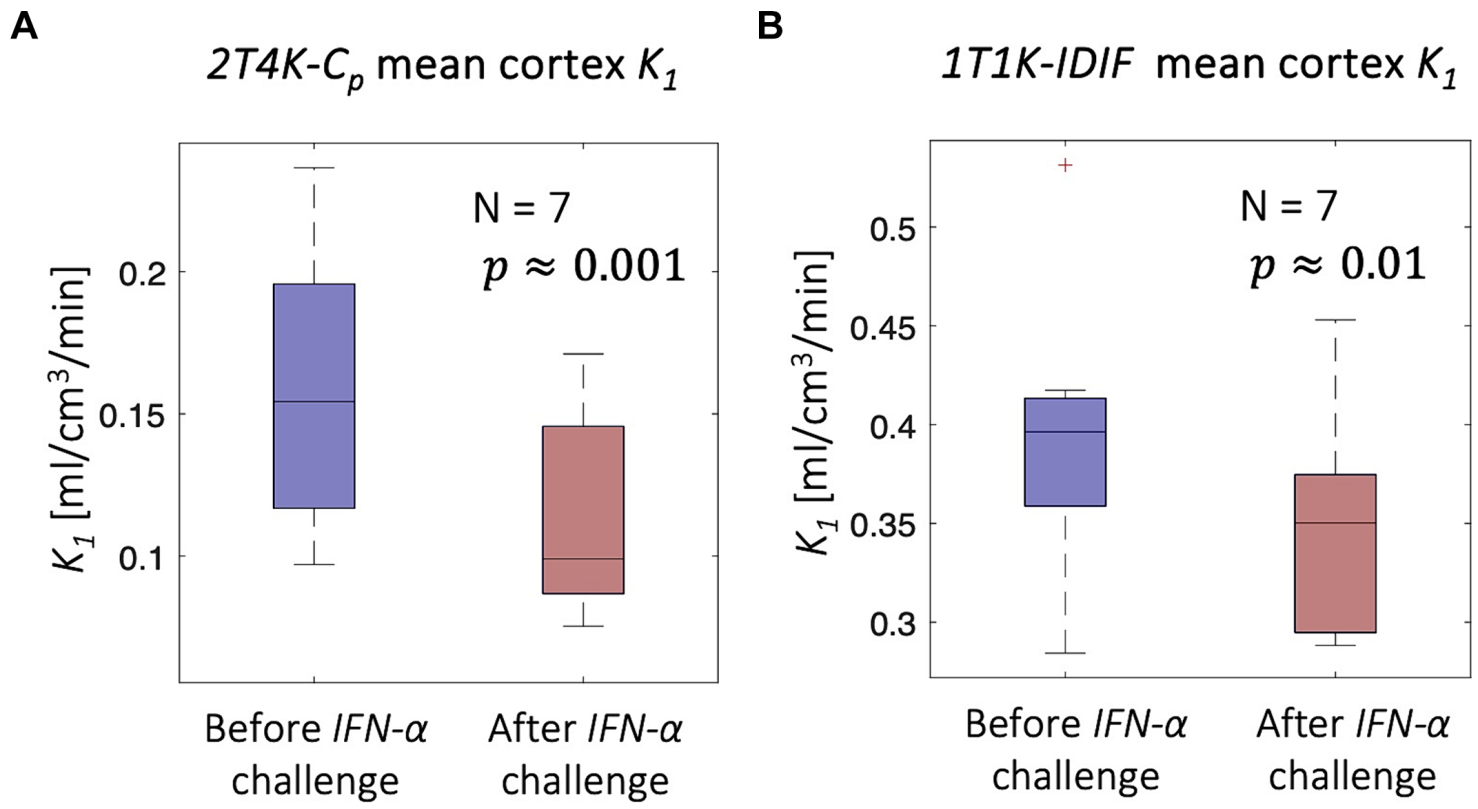
*IFN-α* challenge - [^11^C]PBR28 scans. Figure shows a significant reduction in the mean cortex *K_1_* associated with peripheral inflammation induced with *IFN-α* challenge; the significant reduction is unveiled both in terms of *2T4K-C_p_ K_1_* (panel **A**) and *1T1K-IDIF K_1_* (panel **B**).

Despite the limitations of *1T1K-IDIF* methodology in providing an absolute estimate of the *K_1_* parameter, consistent with previous results, comparisons of mean cortex *K_1_* estimates before and 24 h after the injection of *INF-α* revealed a significant difference in terms of both *2T4K-C_p_* (paired *t* = 5.61; *p* = 0.001) and *1T1K-IDIF* (paired *t* = 3.42; *p* = 0.01) estimates. Figure 8 shows the distribution across subjects of average cortex *K_1_*. The higher inter-subject variability of *1T1K-IDIF* with respect to *2T4K-Cp K_1_* estimates hampers the identification of group effects, thus resulting in lower statistical power when comparing the *K_1_* parameter in physiological on peripherally inflamed conditions (for *2T4K-C_p_*: Cohen’s d effect size = 1.08 and relative difference = −29% *K_1_* estimates; for *1T1K-IDIF*: Cohen’s d effect size = 0.75 and relative difference = −13%).

### 3.4 Test–retest reproducibility of *K_1_* estimates-[^18^F]DPA714 scans

**Figure 9:**
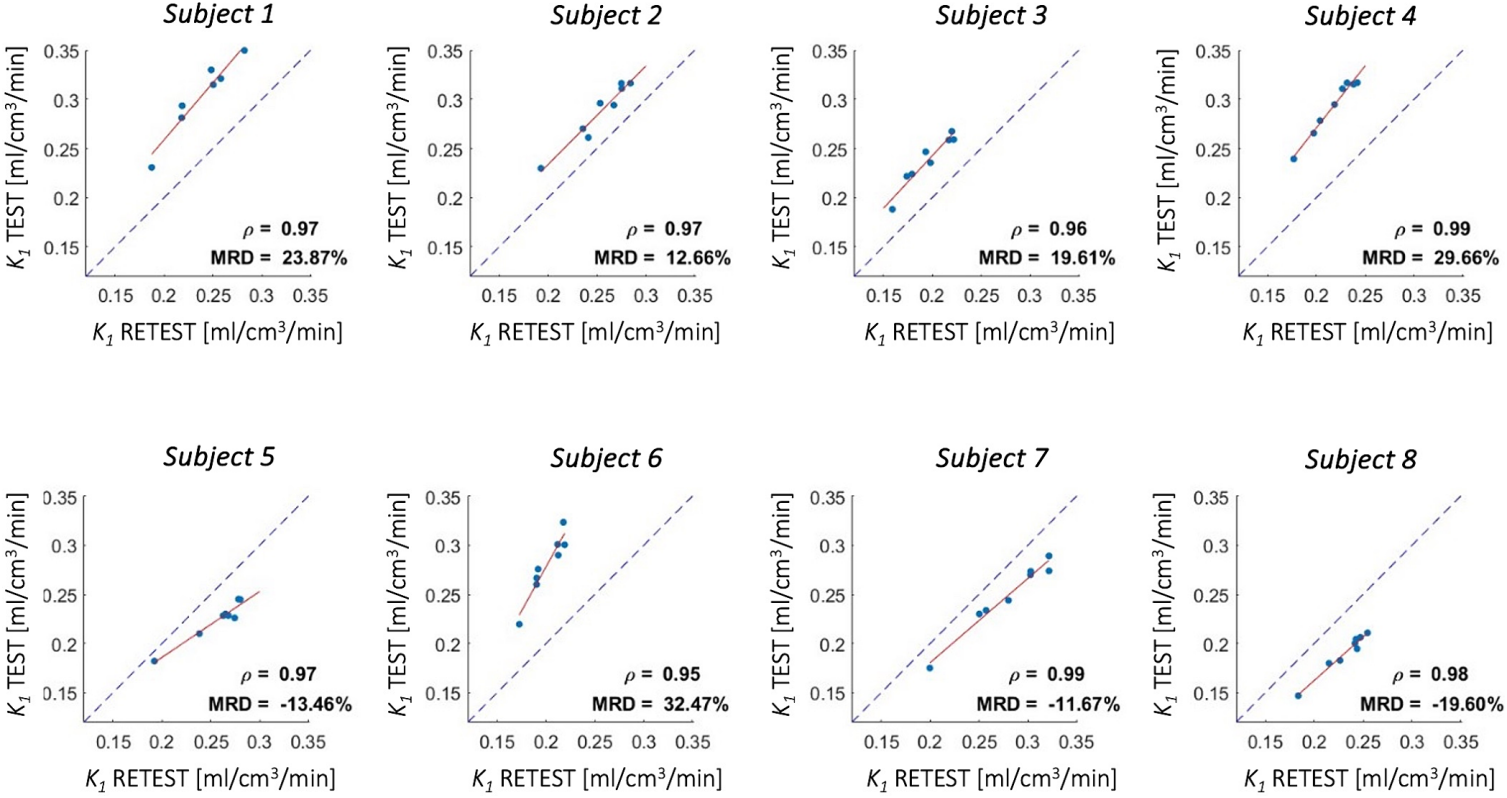
Test–retest reproducibility of *K_1_* estimates - [^18^F]DPA714 scans. Figure shows correlation between *K_1_* estimates computed from test and retest scans for 4 cortical lobes, insula, cerebellum, thalami, and brainstem (red solid line: regression line; gray dotted line: identity line); Pearson’s correlation coefficient (*ρ*) and mean root distance (*MRD*) between test and retest estimates are reported in each panel.

Good-to-high reproducibility of *1T1K-IDIF K_1_* estimates was reported for each of the test–retest subjects (Figure 9), with a Pearson’s Correlation coefficient of 0.97 ± 0.01. *MRD* between estimates ranged from −19.60 to 32.47%.

## 4 Discussion

### 4.1 Model validation

The proposed method represents a completely non-invasive quantification approach for the *K_1_ PET* imaging parameter via the fitting of the early phase of the tracer dynamic. We tested our method on three independent *TSPO PET* datasets. The validation by comparison with the reference-standard blood-based estimates showed high intra-subject correlations between the *2T4K-C_p_* and the *1T1K-IDIF K_1_* estimates with each of the three tested input functions. On the other hand, while a reduced bias was reported when comparing *1T1K-C_p_* and *1T1K-C_b_* to *2T4K-C_p_ K_1_* estimates, high and variable bias was reported for the *1T1K-IDIF*, depending on the *IDIF* variability to approximate blood input functions. This resulted in lower values of between-subject correlations when *IDIF* was adopted as input function of the model. Despite this, the *1T1K-IDIF* estimate allowed us to replicate the significant reduction in the *K_1_* values associated with peripheral inflammation after the immune challenge *IFN-α* (Nettis et al., 2020; Turkheimer et al., 2021).

These results suggest that:

1. *1T1K-IDIF* can be used to reliably measure topological differences in tracer delivery within an individual brain. Practically, this approach has the potential to map subtle changes in the *BBB* affecting individual patients and even single brain lesions within those patients, presenting particularly relevant applications in conditions such as multiple sclerosis.
2. *1T1K-IDIF* can be used to quantify between-subjects differences in terms of tracer delivery, although the high bias makes it unusable for absolute quantification. Our results demonstrated the ability of the non-invasive *1T1K-IDIF* method to unveil alterations in tracers’ blood-to-brain delivery following *IFN-α* challenge, despite the higher variability in *K_1_* estimates and lower sensitivity compared to full blood-based quantification.
3. Given the similarity of performances between *1T1K-C_p_* and *1T1K-C_b_* with the reference standard, the most penalizing assumption for *1T1K-IDIF* is the use of *IDIF* as a proxy of blood input (*Hypothesis #3*), rather than those on simplified modeling (*Hypothesis #1*) or negligible metabolites (*Hypothesis #2*).

### 4.2 Interpretation of *K_1_* measures

According to the Fick principle and Renkin-Crone model, the blood-to-brain delivery rate *K_1_* depends on blood perfusion and the product of capillary permeability and capillary surface area (Renkin, 1959; Crone, 1963). Previous studies have suggested the potential use of the influx rate constant *K_1_* as a biomarker for alterations of brain barrier permeability that are associated with acute or chronic inflammation. In a group of 7 healthy volunteers, increased serum peripheral C-reactive protein, induced by the injection of *IFN-α*, was associated with reduced brain barrier permeability measured by the rate constants *K_1_* of the *TSPO* tracer [^11^C]PBR28 (Nettis et al., 2020; Turkheimer et al., 2021). In a dataset of dynamic [^11^C]PBR28 scans from 11 patients with small vessel disease, kinetic modeling also showed a reduction when comparing white matter hyperintensities to normal appearing white matter (Wright et al., 2020).

Although Sander et al. (2021) showed no significant changes in [^11^C]PBR28 radiotracer kinetic associated with alterations in cerebral blood flow, this evidence is not sufficient to exclude the influence of cerebral blood perfusion on TSPO tracers delivery and uptake. Study by Mackowiak et al. (1998) showed alterations in cerebral perfusion, with a focal increase in the basal ganglia after *IFN-β*, while a decreased level of regional blood perfusion was reported in patients treated for hepatitis with *IFN-α* (Tanaka et al., 2006). It is thus not possible to disentangle an effect of blood perfusion rate alterations from alterations more related to endothelial status of *BBB* and its permeability. Pharmacological studies with perfusion-manipulating interventions could help to provide more biologically specific insights on the interpretation of *K_1_* changes measured with *TSPO PET* tracers.

Importantly, variations in *K_1_* estimates are reflected in the calculations of binding parameters, such as the distribution volume *V_T_* and other kinetic modeling macro-parameters. These variations may be misinterpreted as changes in *TSPO* density. In fact, changes in tracer blood-to-tissue transport have been shown to cause up to 77% of the variation in *V_T_* measures in the case of [^11^C]PBR28 *PET* data (Nettis et al., 2020). When blood data are available and more robust quantifications are possible, such as the full blood-based compartmental modeling used for *V_T_* quantification, the *K_1_* parameter can be directly computed with minimal bias by fitting the standard *2T4K* model. However, when the blood input function is not available and simpler semi-quantitative measures of *TSPO* binding, such as the standardized uptake value (*SUV*) are adopted, the *1T1K-IDIF* methodology can add value by providing a non-invasive measure of tracer influx and allowing the correction of the *SUV* parameter for differences in tracer delivery.

### 4.3 Generalizability of the method

The method was tested on two second-generation *TSPO PET* tracers and data collected both on healthy volunteers and patients (psychiatric disorders, chronic pain), in three different PET centers using different scanners and acquisition protocols. In the case of TSPO PET imaging, the application of *1T1K-IDIF* approach would provide information on tracers delivery on top of neuroinflammation measures. However, the proposed methodological framework has the advantage of being potentially generalizable to other families of tracers - provided the previously described model hypotheses are respected and the method is validated for each new tracer, acquisition protocol, and scanner - especially in the case of radiotracers not actively transported in or out of brain (Tournier et al., 2011). In the case of tracers with relatively high *BBB* permeability and extraction fraction (e.g., β-amyloid PET imaging), the *K_1_* parameter could potentially be adopted as a proxy of cerebral blood flow.

### 4.4 Methodological considerations and study limitations

#### 4.4.1 Blood data

Different blood sampling and analysis procedures were adopted across the three *PET* centers, thus introducing differences in available blood data for the different datasets under study. Firstly, while in the case of *KCL* scans, both *C_b_* and *C_p_uncor_* curves (in addition to *C_p_*) were provided, thus allowing an investigation of both *POB* and *PPf* effects when using the *C_b_* instead of traditional *C_p_* as input function of the reduced model, only the *C_p_uncor_* was available for the *MGH* and *ICM* datasets. Additionally, tracer *fp* - (which is very low for *TSPO* ligands and difficult to measure Turkheimer et al., 2015), thus discouraging several PET imaging centers from its measurement - was solely available for *KCL* data; for this reason, the impact of plasma proteins on blood tracer activity was not taken into account in this study. Finally, in the case of *MGH* and *ICM* data, for which a continuous blood sampling was not performed, temporal resolution of blood and plasma concentration curves was relatively low, potentially affecting the accuracy of model fitting.

#### 4.4.2 Image derived input function

Operatively, the main challenge in the application of the method was linked to the extraction of a reliable input function from the tracer dynamic *PET* images. In our analysis the *AUCratio* acts as a measure of goodness of performance for *IDIF* extraction: the *AUC* approaches 1 as accuracy improves, while worse results are reflected by lower *AUCratio* values, since the *IDIF* approach tends generally to underestimate the blood kinetic peak.

An accurate description of the activity peak in the early phase of the tracer blood kinetic was not trivial, due to the relatively low spatial and temporal resolution in PET dynamic data, which caused, respectively, relevant partial volume effects (*PVEs*), and the impossibility of following the fast rising and descending part of the curve (Volpi et al., 2023), thus introducing variable bias across subjects. In this study no correction for *PVEs* was applied to computed *IDIF*. To the best of our knowledge, there is not a current standard in the application of *PVEs* approaches (Volpi et al., 2023) and effectiveness of these methods strongly depends on image quality, tracer kinetics, and brain anatomy, leading to uncertainty in the corrected *IDIF* and the risk of over-correcting and thus obtaining artificially inflated or distorted concentration time curves. These effects can ultimately result in biased estimates of kinetic parameters as well. Further analyses and a robust validation would be necessary to select the optimal method of *PVEs* correction (depending on scanner and imaging protocols), assess the reliability of the corrected *IDIF*, and evaluate the impact of correction on study outcomes. These two objectives were beyond the aim of this study.

The strong correlation between bias and *AUCratio* suggests how the use of more sophisticated pipelines for vessel segmentation, blood voxels selection and correction for *IDIF* extraction could possibly enhance the performance of the proposed method. The introduction of new generation *PET* scanners, with better sensitivity, improved spatial resolution and temporal resolution, and larger scanner field of view, opens the promise of a high enhancement in the performances of *IDIF* extraction (Volpi et al., 2023). It should be noted that *K_1_* bias in the case of *MGH PET* scans appears less correlated to *AUCratio*. This could be linked to different reasons. Firstly, it is worth mentioning that the *AUCratio*, here assumed as a measure of goodness of *IDIF* approximation of the arterial *C_p_*, simply counts for differences in the area under the two curves, but not for differences in curves shapes. Secondly, *IDIF* extraction and time resolution are dependent on *PET* experimental protocol over which we did not have control being the study performed over pre-existing data. Moreover, as previously highlighted, the blood protocols which will affect the gold standard *K_1_* estimation are different for the sites.

In general, the use of a limited time window after tracer injection has the strong advantage of allowing to reasonably ignore the metabolite production, which, so far, represents the main unresolved issue and one of the main obstacles to a wider application of *IDIF* approach (Zanotti-Fregonara et al., 2011a). The radio-metabolites activity in the late phase of blood *TACs* is indeed known to strongly affect the estimation of tracer binding parameters (Volpi et al., 2023). In a pilot study conducted on [^11^C]PBR28 *PET* scans from *MGH* (*Dataset3*), *V_T_* estimates obtained with the adoption of the *IDIF*, without any correction for metabolites activity, showed high inconsistency (Pearson’s coefficient *ρ* = 0.35) with standard *V_T_* estimates obtained with arterial input function (Whitehead et al., 2023).

#### 4.4.3 Fitting time window

The time window for *1T1K* model fitting was experimentally chosen for each dataset and tracer, considering the kinetic of the tracer, tracer metabolism, and the temporal resolution of the dynamic *PET* scan that would determine the total number of samples used for model fitting for a fixed time window. To note, the relatively low time resolution of tissue *TACs* in the early phase following tracer injection represents one of the major limitations in this study, strongly affecting the accuracy of model fit and *K_1_* estimates. Sensitivity analysis showed robustness of model performances with respect to the adopted fitting-time-window (Supplementary Figures 2, 3), although higher between-subjects correlation for *K_1_* estimates was obtained with longer time windows. Of note, the longer the window and the higher the number of data samples adopted for model fitting and thus the precision of the *K_1_* estimates (i.e., lower coefficient of variation). In the case of [^18^F]DPA714, the adoption of an excessively short time window (3.5 or 4.5 min) resulted in a strong reduction of between-subjects correlation of *1T1K-IDIF* with respect to *2T4K-C_p_ K_1_* estimates.

#### 4.4.4 Comparison of different modeling choices

The analysis of the impact of *V_b_* parametrization on model performance showed modest to no significant difference in *K_1_* estimates but a slight improvement in the precision of estimates in the case the *V_b_* parametrization was included (Supplementary Figure 4). This assumption should be further confirmed for those tissues with a higher blood volume fraction. Also, the minor effect of setting the *V_b_* to 0 could be the consequence of the poor fit of the *V_b_* parameter, due to the poor timing resolution at the early phase of tissue tracers kinetic (e.g., initial frame length of 15 s in the case of *KCL* scans). Interestingly, comparisons of results obtained with different modeling choices (different input functions and compartmental models, both reversible and irreversible) showed better data fits, as assessed by the Akaike Information Criterion (Golla et al., 2017), for *1T2K* compared to *1T1K* model, but at the cost of higher variability, as well as higher bias and less consistency (in terms of both intra- and inter-subjects correlations) to the standard *2T4K-C_p_ ROIs K_1_* estimates (Supplementary Figure 5).

#### 4.4.5 Tracer and scanner effects

In this study, results obtained for [^18^F]DPA714 seemed to demonstrate different performances with respect to [^11^C]PBR28 in terms of bias and intra- and between-subjects correlation of *K_1_* and *R_1_* estimates. The higher *AUCratio* and lower relative bias between *1T1K-IDIF* and *2T4K-C_p_ K_1_* in the *ICM* cohort may be explained by the higher spatial resolution of dynamic *PET* images, with the consequent reduction of the partial volume effects, and the relative slow bolus injection causing the arterial peak to be smoother. Of note, a smaller number of [^18^F]DPA714 scans, with respect to [^11^C]PBR28 scans, were included in the study. Additionally, the acquisition of data in different *PET* centers and scanners introduces relevant batch effects (Joshi et al., 2009), which could result for example in a different level of noise on the image.

Independently from the specific model adopted, differences across sites were also reported in *ROIs K_1_* estimates derived from the quantification of *KCL* and *MGH* [^11^C]PBR28 scans. Supplementary Figure 6 shows how between-subjects pairwise correlations of *ROIs 2T4K-C_p_ K_1_* estimates of healthy controls of *Dataset 1* (*KCL*) and *Dataset3* (*MGH*) are high when comparing subjects from the same dataset and lower when comparing subjects from different datasets, thus revealing differences in terms of *K_1_* topological patterns across the two sites. However, it is important to note that the same pattern reported for *2T4K-C_p_* is detected by *1T1K-IDIF K_1_* estimates. The investigation of the causes of this disagreement is beyond the purposes of this study and not necessarily uniquely related to scanner differences. It is worth mentioning indeed that a significant difference in healthy controls’ age (Wilcoxon rank-sum test *p* < 10^−4^) was found between the two datasets, which could partially explain differences in *K_1_* values between the two scanners.

In general, no effort for data harmonization was performed across the three sites, reflecting the aim of the study to test the performance and general applicability of the proposed methodology for the quantification of *K_1_* on datasets with different characteristics and experimental settings, rather than performing a comparison between sites. However, the specific dependency of the results on tracers, scanners (resolution, sensitivity, noise on data), and acquisition protocols was not systemically investigated. Nonetheless, we managed to provide good preliminary evidence in support of the *1T1K-IDIF K_1_* methodology. Further studies including [^11^C]PK11195 and third-generation *TSPO* tracers (e.g., [^18^F]LW223 (MacAskill et al., 2021) and [^11^C]ER176; Ikawa et al., 2017) would shed additional light on the relationship between method performance and tracers pharmacokinetic.

## 5 Conclusion

Our results validated the use of the *1T1K-IDIF* method as a blood-free alternative for assessing blood-to-brain tracer exchange in *TSPO PET* studies and exploring topological differences in blood–brain-barrier permeability at an individual level.

The proposed method has the main advantage of overcoming the practical limitations of standard compartmental modeling quantification and could potentially be applied to a different family of tracers after proper validation.

The dependence of *K_1_* bias on *IDIF* variability to describe the arterial blood input function suggests that adopting more sophisticated *IDIF* extraction pipelines, in combination with the current development of new generation highly performant *PET* scanners, could potentially lead to a significant improvement in the *1T1K-IDIF* performance.

## Data availability statement

The data analyzed in this study is subject to the following licenses/restrictions: data were used under license for the current study and are not publicly available. Requests to access these datasets should be directed to MV, mattia.veronese@unipd.it.

## Ethics statement

The studies involving humans were approved by local research ethics committee, including the Queen Square London Ethical committee, ref. 16/LO/ 1,520, the Partners institutional review board (IRB) and the radioactive drug research committee (RDRC) at Massachusetts General Hospital (MGH), Boston, MA, and the Medical Bioethics Committee of Ile de France Region. The studies were conducted in accordance with the local legislation and institutional requirements. The participants provided their written informed consent to participate in this study.

## Author contributions

LM: Conceptualization, Methodology, Validation, Visualization, Writing – original draft, Writing – review & editing, Formal analysis. MC: Validation, Visualization, Writing – review & editing, Formal analysis. LB: Writing – review & editing. ES: Writing – review & editing, Methodology. AB: Writing – review & editing, Methodology. JS: Writing – review & editing. MN: Writing – review & editing. VM: Writing – review & editing. OH: Writing – review & editing. FT: Writing – review & editing. MB: Writing – review & editing. BB: Writing – review & editing. BS: Writing – review & editing. ML: Writing – review & editing. MV: Writing – review & editing, Methodology, Supervision, Writing – original draft.

## Footnotes

^1^ *http://Invicro.org*

^2^ *http://www.fil.ion.ucl.ac.uk/spm*

^3^ *http://www.fsl.fmrib.ox.ac.uk/fsl*
