# Supplementary figures and images for: A blood-free modeling approach for the quantification of the blood-to-brain tracer exchange in TSPO PET imaging
Source: Front Neurosci. 2024 Jul 22;18:1395769. doi: 10.3389/fnins.2024.1395769 (PMC11299498; doi:10.3389/fnins.2024.1395769)

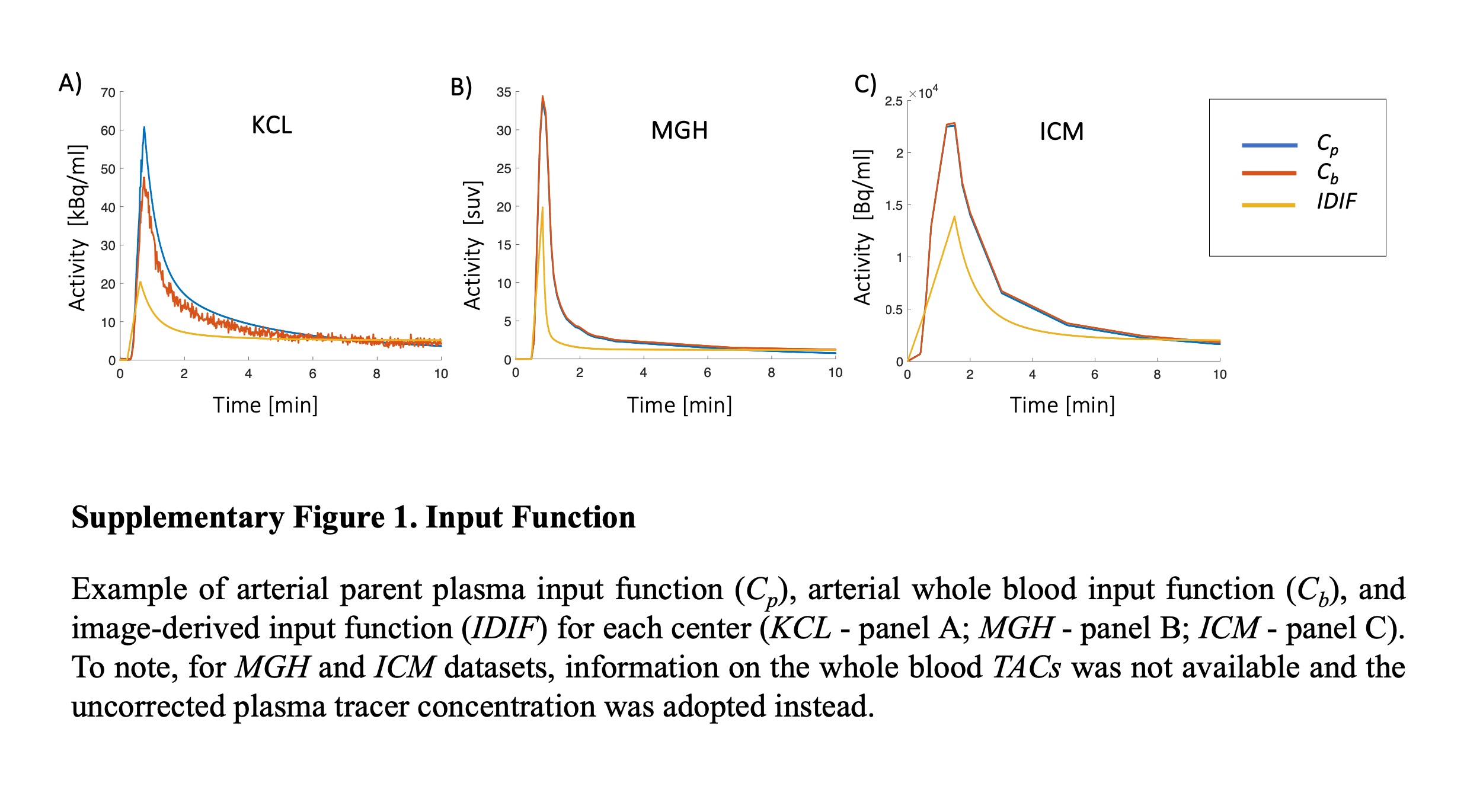

Supplement: Supplementary file 1 [file Image_1.JPEG]

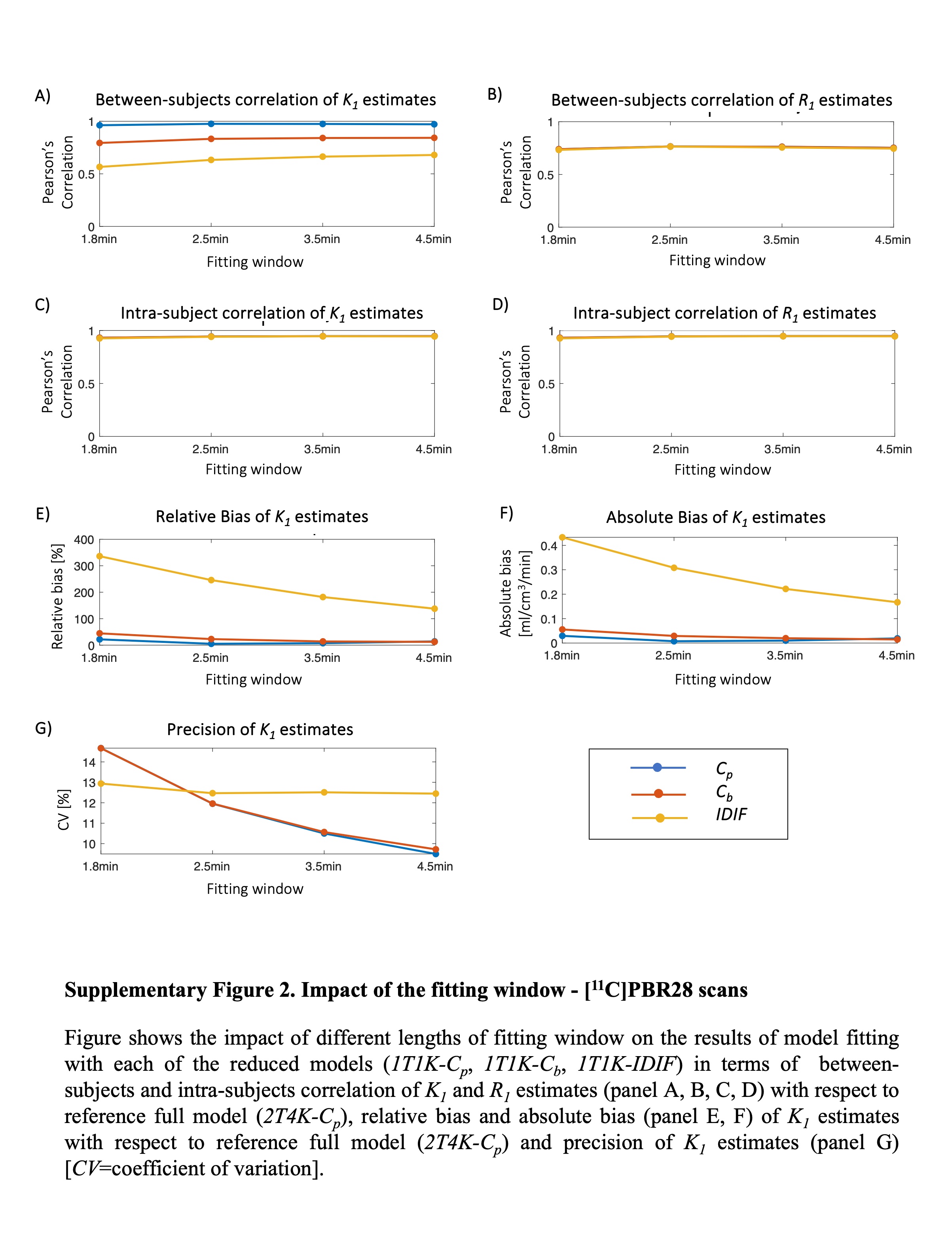

Supplement: Supplementary file 2 [file Image_2.JPEG]

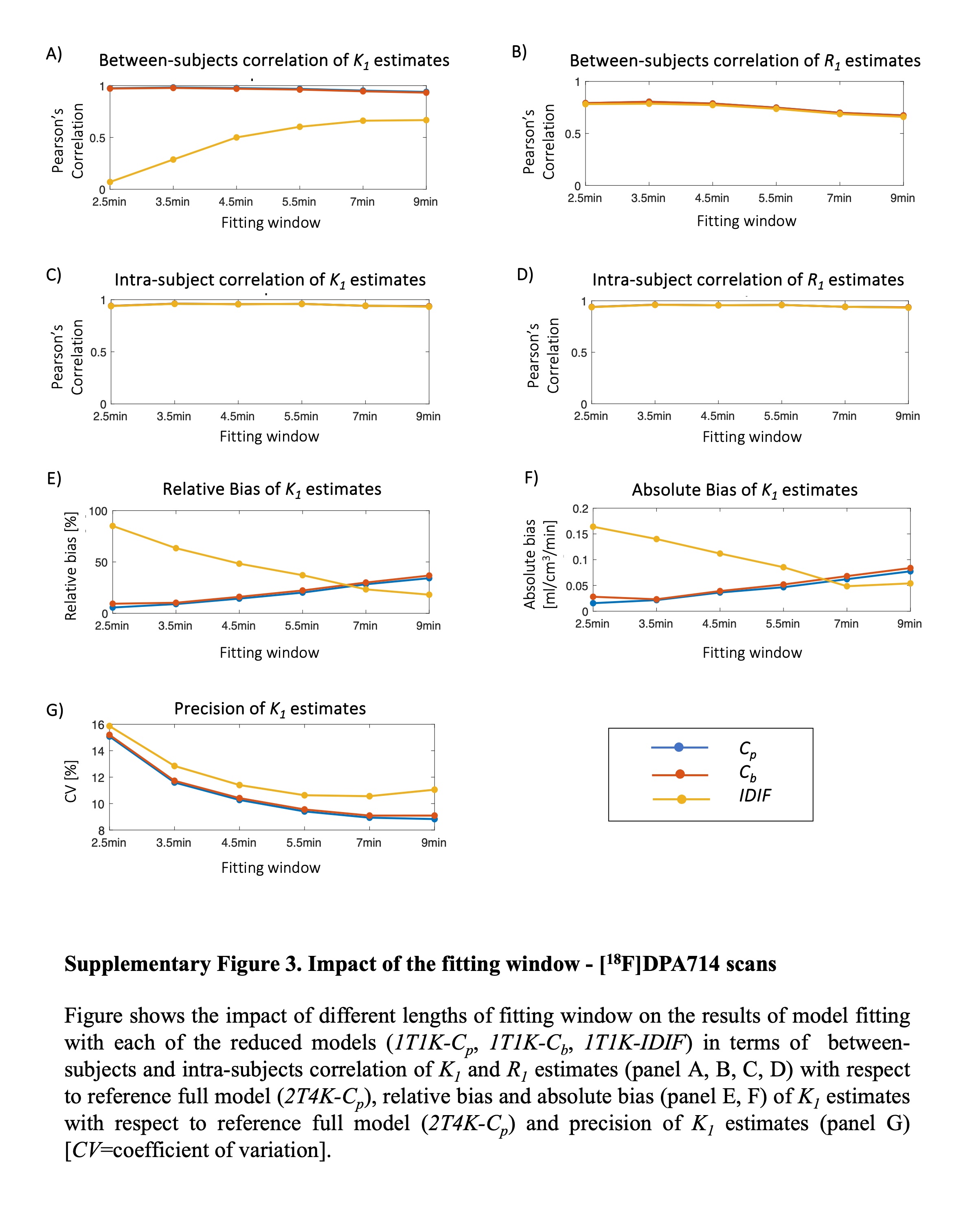

Supplement: Supplementary file 3 [file Image_3.JPEG]

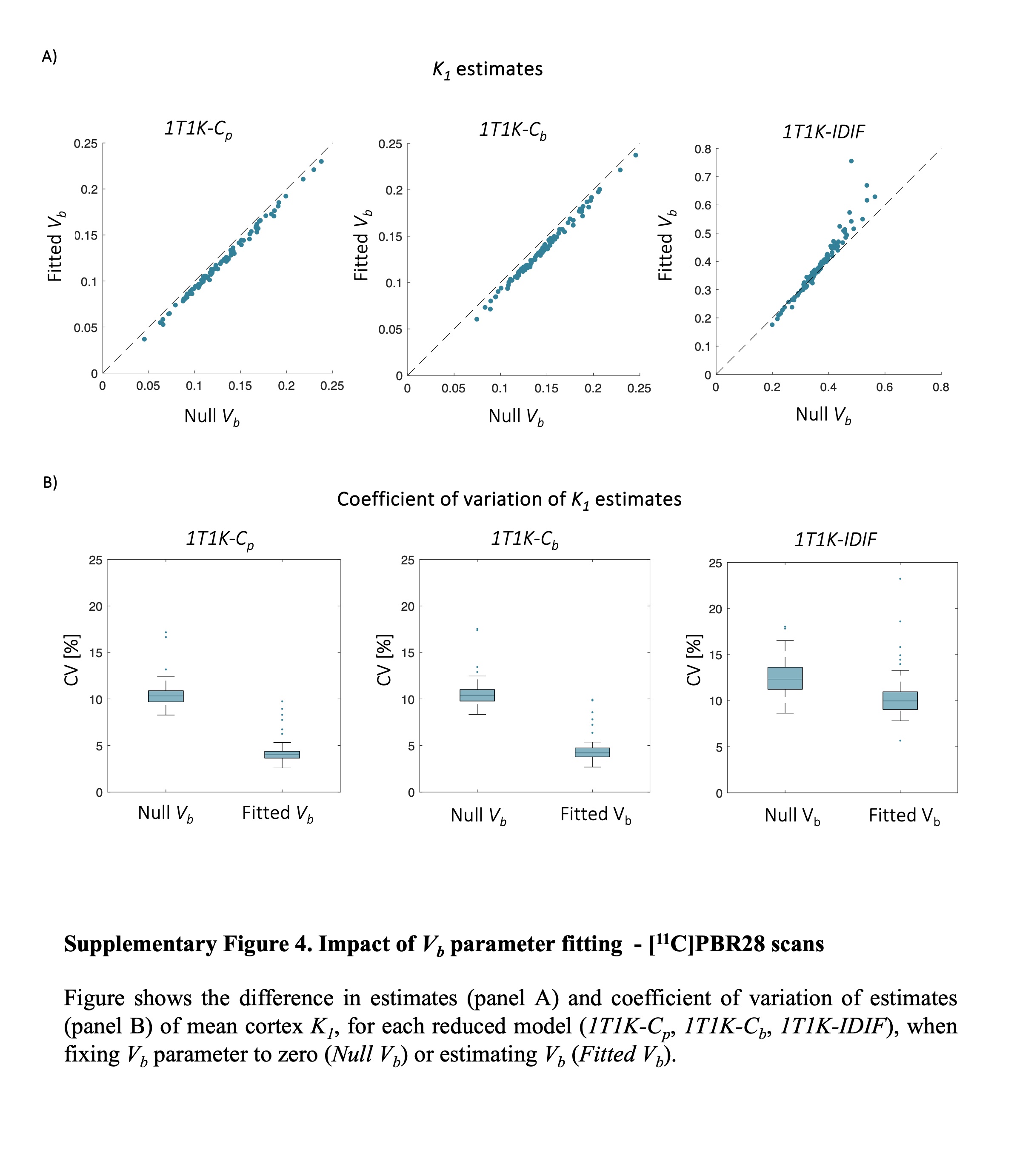

Supplement: Supplementary file 4 [file Image_4.JPEG]

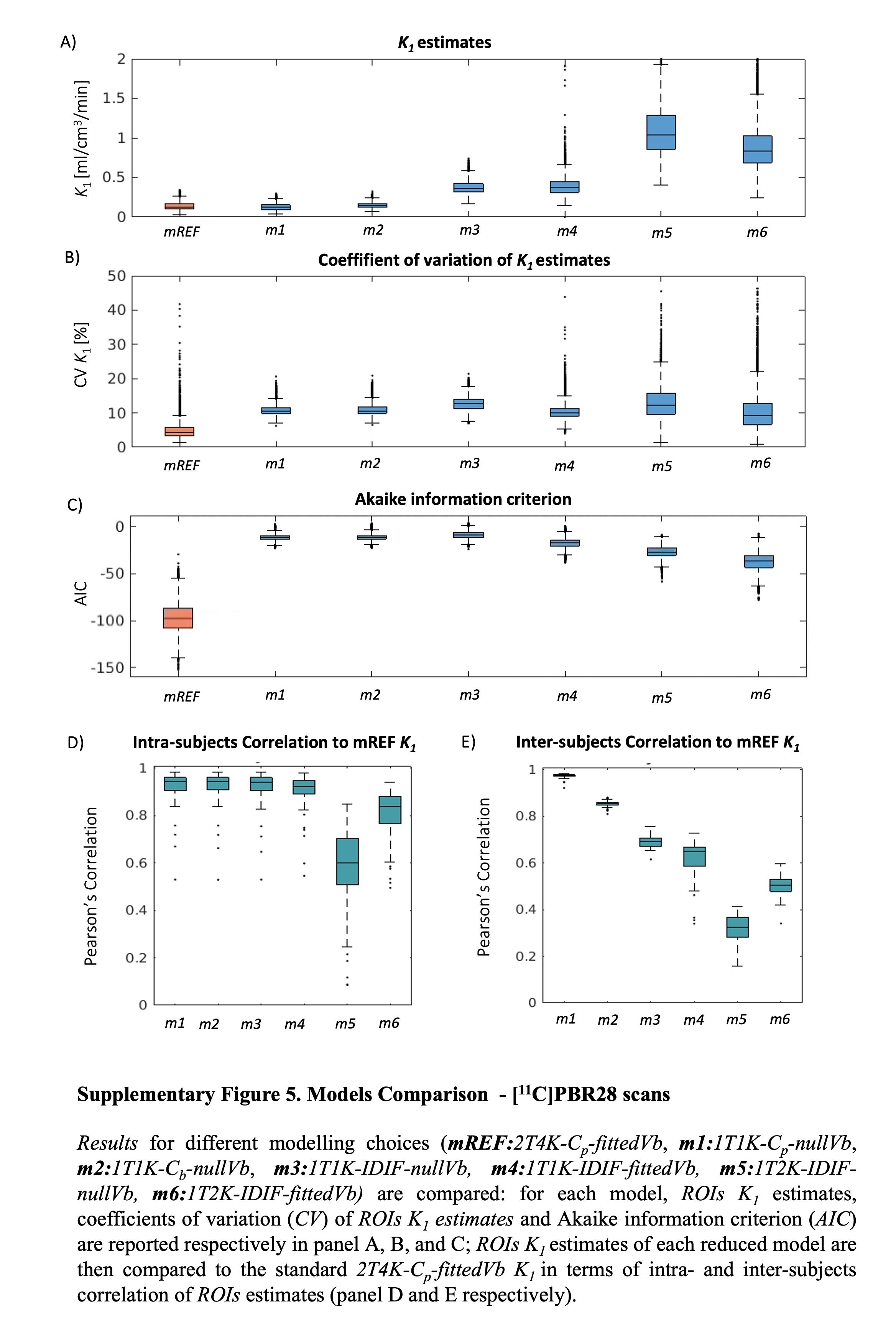

Supplement: Supplementary file 5 [file Image_5.JPEG]

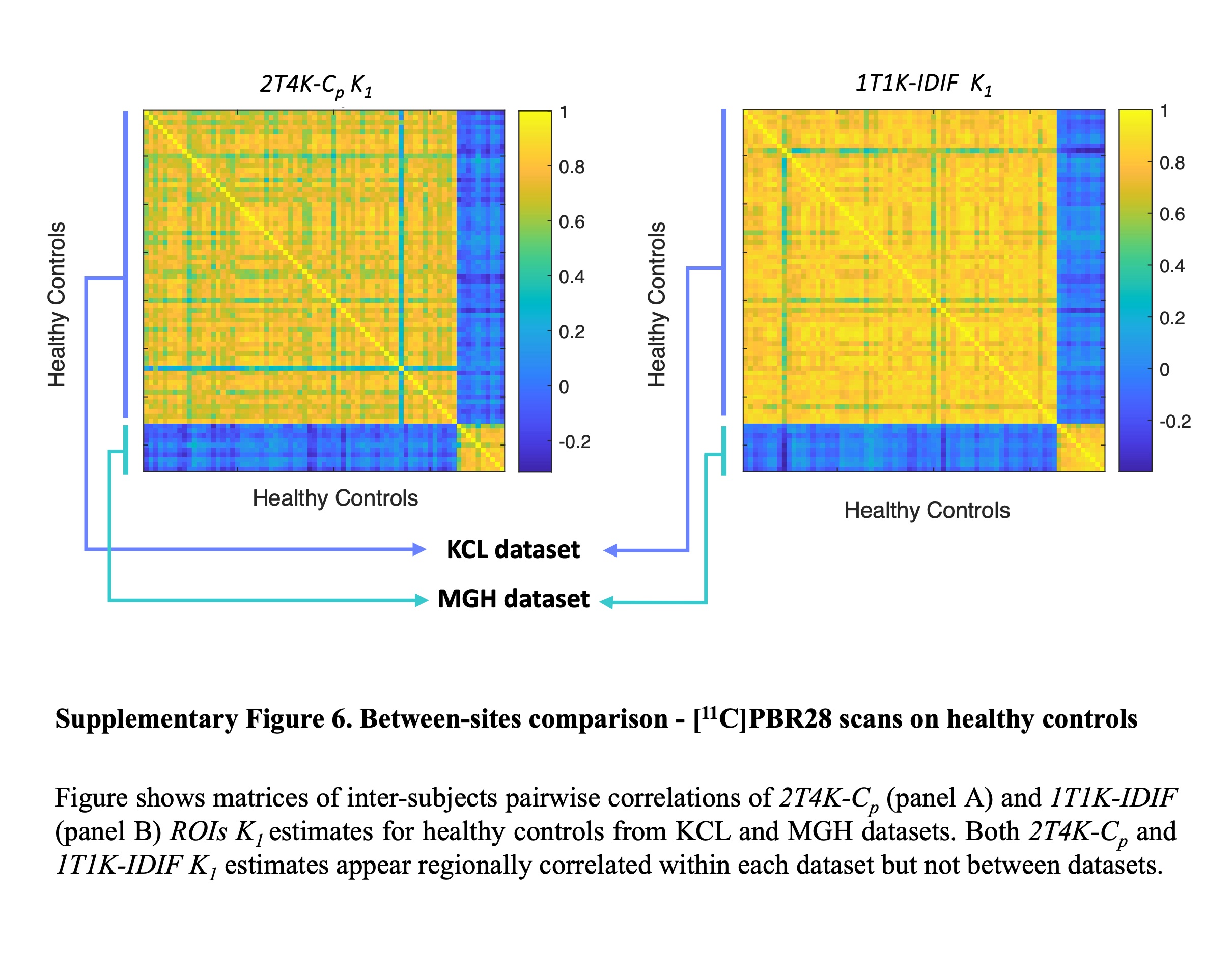

Supplement: Supplementary file 6 [file Image_6.jpg]
